# Efficacy and safety of novel non-steroidal mineralocorticoid receptor antagonists in diabetic kidney disease: an updated systematic review and meta-analysis of randomized controlled trials

**DOI:** 10.3389/fphar.2026.1983608

**Published:** 2026-10-28

**Authors:** Zhonghua Tian, Yuxia Li

**Affiliations:** 1 Department of Pharmacy, Chongqing Traditional Chinese Medicine Hospital, Chongqing, China; 2 Department of Nuclear Medicine, Chongqing Emergency Medical Center, Chongqing, China

**Keywords:** apararenone, diabetic kidney disease, esaxerenone, finerenone, meta-analysis

## Abstract

**Aims:**

To evaluate the efficacy and safety of novel non-steroidal mineralocorticoid receptor antagonists (nsMRAs) in diabetic kidney disease (DKD).

**Methods:**

Following PRISMA 2020, we searched four databases to 31 July 2026, for randomized controlled trials (RCTs) comparing nsMRAs with placebo in adults with DKD. Pooled estimates used random-effects models, with mean differences (MDs) for continuous outcomes and odds ratios (ORs) for dichotomous outcomes, each with 95% confidence intervals (CIs). Risk of bias was assessed with Cochrane RoB 2, and certainty of evidence with GRADE.

**Results:**

Eight RCTs (15,143 patients) were included. nsMRAs reduced log-transformed UACR (MD -0.36 log mg/g, 95% CI -0.44 to −0.28; n = 14,512 from 8 trials, predominantly driven by finerenone (5 of 8 trials), with significant drug-type interaction; P < 0.00001) and systolic BP (MD -4.72 mmHg, 95% CI -8.64 to −0.80; n = 7,538 from 3 trials (6 trials reported systolic BP; only 3 were poolable); predominantly driven by FIGARO-DKD; P = 0.02), but increased serum potassium (MD 0.22 mmol/L, 95% CI 0.16 to 0.29; n = 1,468 from 4 trials (6 trials reported serum potassium; FIDELIO-DKD and FIGARO-DKD excluded); P < 0.00001) and induced early eGFR decline (MD -3.01 mL/min/1.73 m^2^, 95% CI -4.46 to −1.56; n = 1,218 from 3 trials; P < 0.0001). Hyperkalemia risk was higher (OR 2.24, 95% CI 1.99 to 2.52; n = 14,734 from 6 trials; P < 0.00001), whereas overall adverse events, eGFR decline, and acute kidney injury did not differ. GRADE certainty was low for UACR, systolic BP, serum potassium, and early eGFR decline, and moderate for hyperkalemia.

**Conclusion:**

In DKD, nsMRAs may reduce UACR and systolic BP. However, UACR was driven by finerenone with significant drug-type interaction, requiring agent-specific interpretation. Systolic BP was driven by FIGARO-DKD. Furthermore, nsMRAs may increase serum potassium and hyperkalemia risk, although the serum potassium analysis excluded two large trials. nsMRAs may also cause early hemodynamic eGFR decline. Notably, this review assessed only surrogate endpoints and safety outcomes. Several outcomes were supported by only 3–4 trials. Given these limitations and low-to-moderate certainty, findings warrant cautious interpretation and risk-benefit assessment.

**Systematic Review Registration:**

https://www.crd.york.ac.uk/PROSPERO/view/CRD420251072300, identifier CRD420251072300.

## Introduction

Diabetic kidney disease (DKD) is a common microvascular complication of type 2 diabetes and a leading cause of chronic kidney disease (CKD) worldwide (Gupta et al., 2023; Li et al., 2021). Without appropriate treatment, DKD patients face a high risk of progression to end-stage renal disease (ESRD) (Thomas et al., 2015). Once ESRD develops, the incidence of cardiovascular disease increases significantly (Gansevoort et al., 2013). Notably, cardiovascular complications have been the leading cause of death in DKD patients (Zheng et al., 2018). According to the International Diabetes Federation (IDF), the global diabetic population is projected to reach 783.2 million by 2045, with 30%–40% expected to develop DKD (Gupta et al., 2023; Sun et al., 2022). The large DKD population, particularly those with ESRD, will undoubtedly impose a substantial economic burden on healthcare systems.

In recent years, while renin-angiotensin-aldosterone system inhibitors (RAASi), sodium-glucose cotransporter inhibitors (SGLT2i), and glucagon-like peptide-1 receptor agonists (GLP-1RA) have significantly improved DKD prognosis, residual risk of progression to ESRD remains (Kidney Disease: Improving Global Outcomes Diabetes Work Group, 2022; Lin et al., 2023; Perkovic et al., 2019).

Numerous studies have confirmed that mineralocorticoid receptor overactivation is a key driver of cardiorenal disease (Agarwal et al., 2021; Barrera-Chimal et al., 2022; Barrera-Chimal et al., 2019; El Mouhayyar et al., 2025; Lother et al., 2015). Novel non-steroidal mineralocorticoid receptor antagonists (nsMRAs), such as finerenone, apararenone, and esaxerenone, have shown outstanding cardiorenal benefits in DKD patients (Bakris et al., 2020; Ito et al., 2020; Wada et al., 2021). Of these, finerenone has been approved by the FDA for DKD and heart failure (Bayer, 2025; Frampton, 2021). Moreover, nsMRAs, together with RAASi, SGLT2i, and GLP-1RA, have been collectively referred to as the “Fantastic Four” in the field of DKD treatment (Mima, 2022).

Currently, although a previous meta-analysis has evaluated the efficacy of nsMRAs in patients with DKD (Jiang et al., 2022), it focused primarily on comparing the efficacy differences among different agents. Moreover, the dose inclusion criteria were inconsistent across agents. For finerenone, only therapeutic doses (10 mg or 20 mg daily) were included, whereas for other nsMRAs such as apararenone and esaxerenone, all dose levels investigated in the original trials were incorporated. Such discrepancies in dose selection may have introduced selective reporting bias. In addition, that study lacked a systematic analysis of safety data and did not apply the GRADE framework to assess the certainty of evidence, which may undermine the validity of its recommendations. More importantly, new high-quality evidence has recently emerged (Tanaka et al., 2025). These gaps may have implications for clinical decision-making. Therefore, we conducted this updated systematic review and meta-analysis to comprehensively assess the efficacy and safety of nsMRAs in DKD patients and to provide robust evidence for clinical practice.

## Materials and methods

This study strictly adhered to the PRISMA guidelines (Page et al., 2021), and a detailed PRISMA checklist is presented in Supplementary Table S1. The study protocol has been registered in the PROSPERO database (CRD420251072300). The original protocol specified a minimum study duration of at least 24 weeks as an inclusion criterion. During literature screening, we identified the ARTS-DN trial (Bakris et al., 2015), the ARTS-DN Japan trial (Katayama et al., 2017), and a phase II trial (Ito et al., 2019), all of which had a follow-up of only 3 months. To avoid excluding these important trials, the PROSPERO record was amended via a major revision to remove the 24-week minimum duration criterion. The PROSPERO record was also amended to prespecify safety outcomes as treatment-emergent adverse events (TEAEs). These amendments were approved on 28 September 2026, and the revised protocol is publicly available in PROSPERO.

### Search strategy

We systematically searched PubMed, Embase, Web of Science, and the Cochrane Library from inception to 31 July 2026, without language restrictions. A combination of MeSH terms and free-text words was used. Detailed search strategies for each database are provided in Supplementary Table S2. To identify additional eligible trials, we manually screened the reference lists of included articles and relevant meta-analyses and searched ClinicalTrials.gov and the WHO International Clinical Trials Registry Platform (ICTRP) for unpublished or ongoing research. Duplicates were removed using EndNote and cross-checked manually. For studies with multiple reports, we consolidated trial-level data, prioritizing the most complete version.

### Inclusion criteria

Studies were eligible for inclusion if they: (a) enrolled adult patients (aged ≥18 years) with type 2 DKD, a urine albumin-to-creatinine ratio (UACR) of ≥30 mg/g, and an estimated glomerular filtration rate (eGFR) of ≥ 25 mL/min/1.73 m^2^; (b) employed a randomized controlled trial (RCT) design; (c) included a placebo arm for comparison with nsMRAs; and (d) reported at least one prespecified efficacy or safety outcome. There was no minimum follow-up duration. Although the original PROSPERO protocol specified a minimum study duration of at least 24 weeks, this criterion was removed via a major revision to include the ARTS-DN trial (Bakris et al., 2015), the ARTS-DN Japan trial (Katayama et al., 2017), and a phase II trial (Ito et al., 2019), all of which had a 3-month follow-up.

### Exclusion criteria

Studies were excluded if they: (a) involved pediatric populations, or adults with type 1 DKD or other uncommon variants; (b) did not include a placebo control; (c) were non-RCT designs (e.g., case reports, observational or retrospective studies, and reviews); (d) were published as conference abstracts; (e) were preclinical (e.g., animal or *in vitro* investigations); or (f) were duplicate publications.

### Study selection

Two investigators (Z. Tian and Y. Li) independently screened the titles and abstracts of all retrieved records and excluded obviously ineligible studies. Subsequently, they independently assessed the full texts of the remaining articles for eligibility. Any disagreements were resolved by discussion. Figure 1 illustrates the overall study selection process.

**FIGURE 1:**
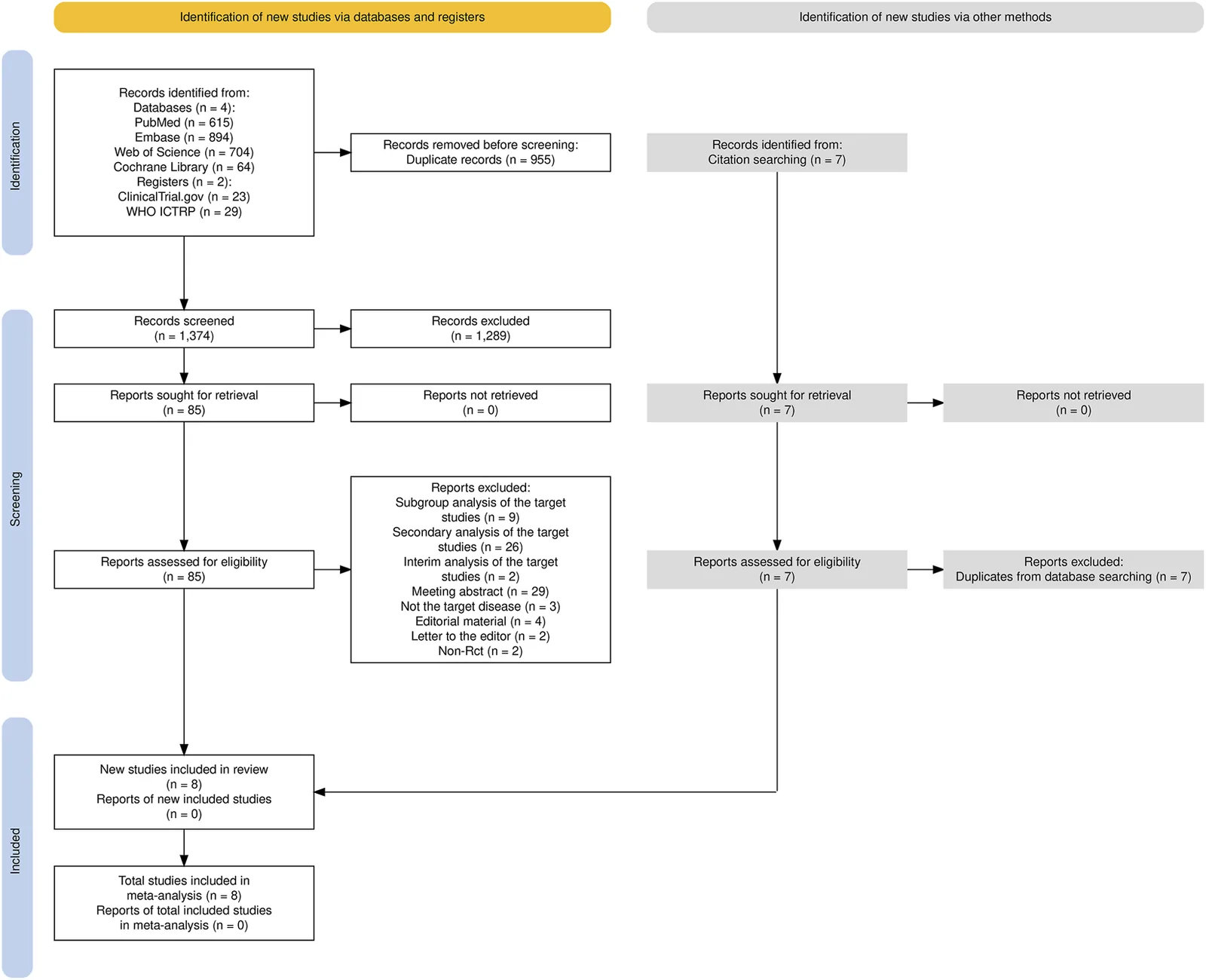
PRISMA flow diagram of the study selection process.

### Data extraction

Data extraction was independently performed by two investigators (Z. Tian and Y. Li). The following information was collected from each included study: (a) study characteristics, including first author, publication year, and study design; (b) baseline characteristics of participants, including sample size, age, gender, UACR, blood pressure (BP), eGFR, serum potassium, and concomitant medications; (c) intervention details, including the agents, dosage regimens, and treatment duration; and (d) treatment outcomes, including changes from baseline in UACR, eGFR, systolic BP, and serum potassium, as well as all TEAEs.

All TEAEs were extracted as reported in each trial, without *post hoc* reclassification. Where original studies did not provide an explicit definition, the outcome was recorded as reported.

For UACR, although the mean follow-up durations of the FIDELIO-DKD trial (Bakris et al., 2020) and the FIGARO-DKD trial (Pitt et al., 2021) were 2.6 and 3.4 years, respectively, both completely reported this parameter only at month 4. Therefore, we extracted the month-4 values for these two studies. For the remaining studies, all continuous outcomes, including the UACR, were extracted as the last reported values, consistent with our prespecified data extraction protocol. Any discrepancies were resolved through discussion.

### Risk of bias assessment

Two investigators (Z. Tian and Y. Li) independently assessed the risk of bias for each trial using the revised Cochrane Risk of Bias 2 (RoB 2) tool (Sterne et al., 2019), covering the following five domains: bias arising from the randomization process, bias due to deviations from intended interventions, bias due to missing outcome data, bias in measurement of the outcome, and bias in selection of reported results. Based on domain-specified ratings, each trial was classified into one of three categories: low risk (if all domains were rated as low risk), some concerns (if any domain raised some concerns), or high risk (if at least one domain was scored as high risk). Any disagreements were resolved by discussion.

## Outcomes

Outcome measures included changes from baseline in UACR, eGFR, systolic BP, and serum potassium, as well as the UACR remission rate.

UACR remission was defined as UACR < 30 mg/g creatinine and a ≥ 30% decrease from baseline at two consecutive time points. This definition was consistent across the included studies. However, the assessment time points differed. Therefore, the original study time points were used, and outcomes were not recoded.

Safety outcomes were prespecified as TEAEs, defined as adverse events that emerged or worsened during treatment, regardless of causality. The prespecified primary safety outcomes were any TEAE, any serious TEAE, and TEAEs leading to discontinuation. Additional safety outcomes, including drug-related TEAEs, specific adverse events of clinical interest (e.g., acute kidney injury [AKI], hyperkalemia, decline in eGFR), and other individual TEAEs, were analyzed as exploratory outcomes. For all safety outcomes, definitions used by the original investigators were extracted verbatim where available, without recoding or secondary classification. Those with identical reported names were combined across studies.

### Statistical analysis

RevMan 5.4. was used to conduct the statistical analyses. Given the likely heterogeneity in clinical and methodological characteristics across included trials, the inverse-variance random-effects model was uniformly employed for all pooled estimates, as recommended by the Cochrane Handbook. For dichotomous outcomes, including the UACR remission rate and incidence of TEAEs, odds ratios (ORs) were calculated. A continuity correction of 0.5 was applied for single zero-event trials, and no trials were excluded for this reason. For continuous outcomes, the analytic approach varied by indicator and data availability. All estimates were reported with 95% confidence intervals (CIs).

For UACR, a log-transformed parameter in the original trials, we extracted the LS mean ratios to baseline as reported for both the intervention and control arms. The treatment effect for each study was then estimated as the ratio of these two ratios, which was natural log-transformed before pooling. These ln-ratios were subsequently combined using the ratio-of-means method (Friedrich et al., 2008), and the combined logarithmic estimate was back-transformed to yield geometric mean ratios (GMRs).

For eGFR and serum potassium, all original studies reported the mean changes from baseline with standard deviations (SDs) and sample sizes. Therefore, these outcomes were analyzed using mean differences (MDs) based on the extracted means, SDs, and sample sizes.

Notably, six trials reported serum potassium outcomes, two of which (Bakris et al., 2020; Pitt et al., 2021) reported changes from baseline but did not provide the corresponding SDs. More crucially, neither the 95% CIs, standard errors (SEs), nor exact P-values were reported, precluding the back-calculation of the SDs. Despite attempts to contact the authors, the original data could not be obtained. Therefore, we applied the correlation-based imputation method recommended by the Cochrane Handbook to impute the missing SDs. Specifically, correlation coefficients (*r*) between baseline and end-of-study measurements were derived from the remaining included studies with complete dispersion data using the bivariate identity: $r=\frac{\left({\text{SD}}_{\text{baseline}}^{2}+{\text{SD}}_{\text{final}}^{2}-{\text{SD}}_{\text{change}}^{2}\right)}{2\times {\text{SD}}_{\text{baseline}}\times {\text{SD}}_{\text{final}}}$ . The mean *r* value was then applied to impute the missing SD_change_ using the formula: ${\text{SD}}_{ch\text{ange}}=\sqrt{{\text{SD}}_{\text{baseline}}^{2}+{\text{SD}}_{\text{final}}^{2}-2\times r_{\text{mean}}\times {\text{SD}}_{\text{baseline}}\times {\text{SD}}_{\text{final}}}.$

Before performing the imputation, we planned to assess the plausibility of imputation by comparing the imputed SDs against the range reported in the other included studies. However, the imputed values exceeded this range, and incorporating them into a sensitivity analysis led to a marked increase in heterogeneity across the pooled estimates. To preserve the robustness and reliability of the primary findings, we therefore excluded these imputed results from the main meta-analysis and confined them to the sensitivity analysis. In addition, we evaluated the impact of this approach by repeating the meta-analysis under three prespecified scenarios: excluding trials with imputed SDs, re-imputing the missing SDs assuming the average *r* decreased or increased by 0.2. The differences in the pooled effect estimates across scenarios were quantified to assess the robustness of the method. Finally, the two studies that originally lacked SD reports were not quantitatively pooled in the primary analysis. Their results were instead described narratively in the main text, and all imputation-based data were kept within the sensitivity analysis.

For systolic BP, six trials reported this outcome, but data availability varied across studies, precluding the inclusion of all six trials in the same pooled synthesis. The FIGARO-DKD trial (Pitt et al., 2021) reported the between-group MD with 95% CIs, from which we derived the SE using standard formulas: $SE=\frac{{CI}_{upper}-{CI}_{lower}}{2\times 1.96}$ . The other two trials (Katayama et al., 2017; Tanaka et al., 2025) reported the change from baseline with SDs and sample sizes for both arms. Using these data, we calculated the SEs using standard formulas: $\text{SE}=\frac{\text{SD}}{\sqrt{n}}$ . The remaining three trials (Bakris et al., 2020; Ito et al., 2019; Wada et al., 2021) reported only the between-group MD without sufficient data to compute the SE (e.g., 95% CIs or exact P-values). Despite attempts to contact the corresponding authors to obtain these unreported variance data, the required statistics could not be retrieved. Moreover, given that these three studies accounted for half of the trials reporting this outcome, any statistical imputation of their missing variance parameters would rely on untestable assumptions and could yield unreliable pooled estimates. Therefore, no imputation was attempted for this outcome. Consequently, only the three studies with available data were pooled, while the remaining three studies were described narratively.

The prespecified aggregate safety outcomes (any TEAE, any serious TEAE, and TEAEs leading to discontinuation) were meta-analyzed and reported with P values. Given the small number of prespecified comparisons, no multiplicity correction was deemed necessary. Analyses of drug-related TEAEs, specific adverse events of clinical interest (e.g., AKI, hyperkalemia, decline in eGFR), and other individual TEAEs were exploratory and hypothesis-generating. These analyses were therefore left unadjusted for multiplicity, and their P values should be interpreted with caution.

Heterogeneity was assessed using the I^2^ statistic and Cochran’s Q test (Higgins and Thompson, 2002), with the conventional thresholds of 25%, 50%, and 75% corresponding to low, moderate, and high heterogeneity, respectively. However, given the small number of studies incorporated, the I^2^ value was deemed to have limited dependability. Consequently, we did not rely exclusively on those fixed cutoffs. Instead, we performed a holistic heterogeneity assessment that integrated numerical metrics with a qualitative inspection of the size and consistency of treatment effects.

To assess the stability of the findings, leave-one-out analyses were conducted for measures that included at least three studies. Additionally, to assess the impact of short-term studies (follow-up duration less than 24 weeks) on the findings, we performed a sensitivity analysis excluding these short-term trials.

Exploratory subgroup analyses were performed to investigate potential sources of heterogeneity and to assess the consistency of treatment effects across different patient and study characteristics. To avoid spurious findings, all subgroup analyses were restricted to comparisons represented by at least two studies, and subgroup definitions were tailored to the number of available studies for each outcome. For the UACR outcome, subgroup analyses were stratified by age (≥ 65 vs. < 65 years), sample size (≥ 1,000 vs. < 1,000 participants), study duration (≥ 6 vs. < 6 months), baseline UACR (≥ 300 vs. < 300 mg/g), baseline eGFR (≥ 60 vs. < 60 mL/min/1.73 m^2^), and drug type (finerenone vs. other nsMRAs). These cutoffs were selected based on established clinical thresholds (e.g., KDIGO classification for UACR and eGFR), previous literature, or clinically meaningful distributions. For the serum potassium outcome, subgroup analyses were conducted for age (≥ 65 vs. < 65 years), sample size (≥ 300 vs. < 300 participants), study duration (≥ 6 vs. < 6 months), and drug type (finerenone vs. other nsMRAs). The sample size threshold differed from that used for UACR because fewer studies contributed to serum potassium and their designs were homogeneous. A uniform threshold would have resulted in subgroups with fewer than two studies. We originally planned subgroup analyses for the serum potassium outcome according to baseline UACR, eGFR, and baseline potassium level. However, these were not performed because only four available studies contributed data to this outcome, and further stratification would have resulted in subgroups with fewer than two studies. Interaction P-values were calculated using the Cochran Q test across subgroups. Notably, due to the exploratory nature of these analyses, results should be interpreted with caution.

Given that only eight RCTs met our inclusion criteria, quantitative tests for publication bias such as funnel plot asymmetry and Egger’s regression were not undertaken. These tests lack adequate statistical power when based on fewer than ten effect estimates (Sterne et al., 2011). To mitigate potential reporting bias, we extended our electronic search to major trial registries (ClinicalTrials.gov and the WHO ICTRP) on 31 July 2026, without imposing restrictions on language, geographic region, or registration date. Our registry search, however, retrieved no records that satisfied the prespecified eligibility criteria after full review.

Throughout the analysis, a two-sided P value of less than 0.05 denoted statistical significance.

### Certainty of evidence

The certainty of evidence for each primary outcome was assessed using the GRADE framework (Guyatt et al., 2011). Given that all primary analyses were based on randomized trials, the initial certainty rating was set to high. We then sequentially evaluated five domains that may lead to downgrading: risk of bias, inconsistency, indirectness, imprecision, and publication bias. For continuous outcomes, we prioritized pre-specified or established minimally important differences (MIDs) from the published literature when available. For UACR, we adopted the guideline-recommended threshold of a 30% reduction (Agarwal et al., 2023; Heerspink et al., 2019; Navaneethan et al., 2025), which corresponds to approximately −0.36 on the natural log scale given that UACR data were usually log-transformed for meta-analysis. For other continuous outcomes without an established MID (e.g., eGFR, systolic BP, and serum potassium), we calculated the default MID as 0.5 × the median of the baseline SDs from the control arms across all included studies, in accordance with the GRADE Working Group recommendations (Schünemann et al., 2022; Schünemann et al., 2013). For dichotomous outcomes, we used the GRADE default MID of 1.25 for harm (or 0.75 for benefit) on the OR scale (Schünemann et al., 2022; Schünemann et al., 2013). These thresholds, along with other factors (e.g., the width of the 95% CI, total sample size, and the distribution of study weights), informed our judgement of imprecision. Finally, each outcome was assigned a final certainty rating of high, moderate, low, or very low.

## Results

### Study selection and characteristics

The initial search identified 2,329 records, including 2,277 from electronic databases and 52 from trial registries. In addition, 7 reports were identified from the reference lists of previous meta-analyses, but all were duplicates of records from the electronic databases. After removing duplicates, 1,374 records underwent title and abstract screening, resulting in the exclusion of 1,289 records based on predefined eligibility criteria. Subsequently, 85 potentially relevant studies were assessed via full-text review. Finally, eight studies (enrolling 15,143 DKD patients) met the inclusion criteria and were included in the final analysis (Bakris et al., 2020; Bakris et al., 2015; Ito et al., 2020; Ito et al., 2019; Katayama et al., 2017; Pitt et al., 2021; Tanaka et al., 2025; Wada et al., 2021). The study selection process is illustrated in Figure 1.

Among the eight included studies, five investigated finerenone (Bakris et al., 2020; Bakris et al., 2015; Katayama et al., 2017; Pitt et al., 2021; Tanaka et al., 2025), two evaluated esaxerenone (Ito et al., 2020; Ito et al., 2019), and one assessed apararenone (Wada et al., 2021). The dosage ranged from 1.25 to 20 mg/d for finerenone, 0.625 to 5 mg/d for esaxerenone, and 2.5 to 10 mg/d for apararenone. Baseline eGFR ranged from 44 to 78 mL/min/1.73 m^2^ and baseline UACR ranged from 101 to 867 mg/g. Follow-up duration ranged from 3 to 54 months. Detailed study characteristics are presented in Table 1.

**TABLE 1:** Baseline characteristics of studies and participants included.

Characteristic	Bakris et al., 2020	Katayama et al., 2017	Pitt et al., 2021	Wada et al., 2021	Bakris et al., 2015	Ito et al., 2019	Ito et al., 2020	Tanaka et al., 2025
Study name	FIDELIO-DKD	ARTS-DN Japan	FIGARO-DKD	NA	ARTS-DN	NA	ESAX-DN	FIVE-STAR
Clinical trial number	NCT02540993	NCT01968668	NCT02545049	NCT02517320	NCT01874431	NCT02345057	JapicCTI-173695	NCT05887817
Research institution	48 countries	16 centers in Japan	48 countries	Japan	23 countries	71 centers in Japan	135 centers in Japan	13 centers in Japan
Study design	A multicenter, randomized, double-blind, placebo-controlled study	A multicenter, randomized, double-blind, placebo-controlled study	A multicenter, randomized, double-blind, placebo-controlled study	A multicenter, randomized, double-blind, placebo-controlled study	A multicenter, randomized, double-blind, placebo-controlled study	A multicenter, randomized, double-blind, placebo-controlled study	A multicenter, randomized, double-blind, placebo-controlled study	A multicenter, randomized, double-blind, active-controlled study
Population characteristics	Patients (aged ≥18 years) had a clinical diagnosis of T2D, with either: UACR ≥30 to <300 mg/g and eGFR ≥25 to <60 mL/min per 1.73m^2^, or UACR ≥300 to ≤5,000 mg/g and eGFR ≥25 to <75 mL/min/1.73m^2^	Patients (aged ≥18 years) had a clinical diagnosis of T2D, with persistent UACR ≥30 and an eGFR ≥30 to <90 mL/min/1.73m^2^	Patients (aged ≥18 years) had a clinical diagnosis of T2D, with either: Persistent UACR ≥30 to <300 mg/g and eGFR ≥25 to <90 mL/min per 1.73m^2^, or persistent UACR ≥300 to ≤5,000 mg/g and eGFR ≥60 mL/min/1.73m^2^	Patients (aged 20-75 years) had a clinical diagnosis of T2D, with UACR ≥50 to <300 mg/g and eGFR ≥60 mL/min/1.73m^2^	Patients (aged ≥18 years) had a clinical diagnosis of T2D, with UACR ≥30 mg/g and eGFR ≥30 to <60 mL/min/1.73m^2^	Patients (aged ≥20 years) had a clinical diagnosis of T2D, with UACR ≥45 to <300 mg/g and eGFR ≥30 mL/min/1.73m^2^	Patients (aged ≥20 years) had a clinical diagnosis of T2D, with UACR ≥45 to <300 mg/g and eGFR ≥30 mL/min/1.73m^2^	Patients (aged ≥18 years) had a clinical diagnosis of T2D, with UACR ≥30 to <3,500 mg/g and eGFR ≥25 to <90 mL/min/1.73m^2^
Intervention measure	Intervention group: Finerenone 10-20 mg/d Control group: Placebo	Intervention group: Finerenone 1.25-20 mg/d Control group: Placebo	Intervention group: Finerenone 10-20 mg/d Control group: Placebo	Intervention group: Apararenone 2.5-10 mg/d Control group: Placebo	Intervention group: Finerenone 1.25-20 mg/d Control group: Placebo	Intervention group: Esaxerrnone 0.625-5 mg/d Control group: Placebo	Intervention group: Esaxerenone 1.25-2.5 mg/d Control group: Placebo	Intervention group: Finerenone 10–20 mg/d Control group: Placebo
Duration of research (months)	48	3	54	6	3	3	12	6
Sample size, no.	Intervention group: 2,833 Control group: 2,841	Intervention group: 84 Control group: 12	Intervention group: 3,686 Control group: 3,666	Intervention group: 220 Control group: 72	Intervention group: 727 Control group: 94	Intervention group: 285 Control group: 73	Intervention group: 222 Control group: 227	Intervention group: 51 Control group: 50
Sex, male, no.	Intervention group: 1953 Control group: 2030	Intervention group: 67 Control group: 10	Intervention group: 2,528 Control group: 2,577	Intervention group: 167 Control group: 54	Intervention group: 570 Control group: 69	Intervention group: 222 Control group: 57	Intervention group: 165 Control group: 180	Intervention group: 34 Control group: 33
Age, mean ± SD, years	Intervention group: 65.4 ± 8.9 Control group: 65.7 ± 9.2	Intervention group: 62.40 ± 9.80 Control group: 66.75 ± 9.02	Intervention group: 64.1 ± 9.7 Control group: 64.1 ± 10.0	Intervention group: 62.3 ± 9.0 Control group: 60.1 ± 8.5	Intervention group: 64.33 ± 9.21 Control group: 63.26 ± 8.68	Intervention group: 65 ± 9 Control group: 66 ± 10	Intervention group: 66 ± 10 Control group: 66 ± 9	Intervention group: 73.0 ± 11.1 Control group: 73.0 ± 10.4
Duration of diabetes, mean ± SD, years	Intervention group: 16.6 ± 8.8 Control group: 16.6 ± 8.8	NA	Intervention group: 14.5 ± 8.6 Control group: 14.4 ± 8.4	Intervention group: 14.38 ± 9.32 Control group: 12.82 ± 7.61	NA	Intervention group: 15 ± 10 Control group: 14 ± 9	Intervention group: 14 ± 8 Control group: 14 ± 9	Intervention group: 14.6 ± 9.3 Control group: 13.0 ± 9.9
HbA1c, mean ± SD, %	Intervention group: 7.7 ± 1.3 Control group: 7.7 ± 1.4	Intervention group: 7.21 ± 0.96 Control group: 7.28 ± 0.72	Intervention group: 7.7 ± 1.4 Control group: 7.7 ± 1.4	Intervention group: 7.21 ± 0.94 Control group: 7.08 ± 0.80	Intervention group: 7.6 ± 1.3 Control group: 7.6 ± 1.3	Intervention group: 6.9 ± 0.6 Control group: 6.9 ± 0.6	Intervention group: 7.0 ± 0.6 Control group: 7.0 ± 0.6	Intervention group: 6.9 ± 0.7 Control group: 6.7 ± 0.7
BMI, mean ± SD, kg/m^2^	Intervention group: 31.1 ± 6.0 Control group: 31.1 ± 6.0	Intervention group: 27.07 ± 4.34 Control group: 26.68 ± 3.24	Intervention group: 31.5 ± 6.0 Control group: 31.4 ± 5.9	Intervention group: 26.70 ± 4.56 Control group: 27.00 ± 4.64	Intervention group: 31.75 ± 5.57 Control group: 32.49 ± 5.27	Intervention group: 25.8 ± 3.8 Control group: 25.9 ± 4.2	Intervention group: 26.2 ± 4.1 Control group: 25.9 ± 4.0	Intervention group: 24.2 ± 3.9 Control group: 24.5 ± 3.7
History of hypertension, no.	Intervention group: 2,737 Control group: 2,768	Intervention group: 80 Control group: 12	Intervention group: 3,544 Control group: 3,517	NA	Intervention group: 685 Control group: 89	Intervention group: 274 Control group: 71	NA	Intervention group: 47 Control group: 45
SBP, mean ± SD, mmHg	Intervention group: 138.1 ± 14.3 Control group: 138.0 ± 14.4	Intervention group: 137.22 ± 15.36 Control group: 135.72 ± 16.90	Intervention group: 135.8 ± 14.0 Control group: 135.7 ± 14.1	Intervention group: 135.9 ± 11.9 Control group: 133.4 ± 11.8	Intervention group: 137.9 ± 14.3 Control group: 139.9 ± 14.3	Intervention group: 138 ± 12 Control group: 138 ± 11	Intervention group: 140 ± 10 Control group: 140 ± 10	Intervention group: 130.0 ± 17.8 Control group: 129.0 ± 17.0
DBP, mean ± SD, mmHg	Intervention group: 75.8 ± 9.7 Control group: 75.8 ± 9.7	Intervention group: 77.18 ± 10.91 Control group: 69.25 ± 11.63	Intervention group: 76.8 ± 9.5 Control group: 76.8 ± 9.6	Intervention group: 77.9 ± 9.9 Control group: 77.7 ± 9.1	Intervention group: 77.0 ± 9.8 Control group: 78.2 ± 9.7	Intervention group: 76 ± 9 Control group: 76 ± 9	Intervention group: 83 ± 8 Control group: 84 ± 8	NA
eGFR, mean ± SD, ml/min/1.73 m^2^	Intervention group: 44.4 ± 12.5 Control group: 44.3 ± 12.6	Intervention group: 65.23 ± 13.70 Control group: 60.88 ± 16.53	Intervention group: 67.6 ± 21.7 Control group: 68.0 ± 21.7	Intervention group: 74.1 ± 20.3 Control group: 77.5 ± 20.5	Intervention group: 66.9 ± 21.8 Control group: 72.2 ± 20.4	Intervention group: 67 ± 17 Control group: 69 ± 19	Intervention group: 69 ± 18 Control group: 69 ± 18	Intervention group: 55.7 ± 14.5 Control group: 58.3 ± 17.9
UACR, mean ± SD/median (range), mg/g	Intervention group: 833 (441-1,628) Control group: 867 (453-1,645)	Intervention group: 205.94 ± 184.09 Control group: 256.80 ± 166.31	Intervention group: 302 (105-749) Control group: 315 (111-731)	Intervention group: 137.74 ± 82.15 Control group: 141.62 ± 88.23	Intervention group: 204.9 ± 181.9 Control group: 188.4 ± 169.8	Intervention group: 109.3 ± 55.0 Control group: 110.0 ± 54.5	Intervention group: 113 (46-286) Control group: 110 (47-278)	Intervention group: 221.8 (67.8-533.1) Control group: 190.9 (76.6-470.9)
Serum potassium, mean ± SD, mmol/L	Intervention group: 4.37 ± 0.46 Control group: 4.38 ± 0.46	Intervention group: 4.18 ± 0.37 Control group: 4.21 ± 0.18	Intervention group: 4.33 ± 0.43 Control group: 4.33 ± 0.43	Intervention group: 4.28 ± 0.28 Control group: 4.24 ± 0.31	Intervention group: 4.30 ± 0.42 Control group: 4.30 ± 0.48	Intervention group: 4.2 ± 0.3 Control group: 4.3 ± 0.3	Intervention group: 4.4 ± 0.3 Control group: 4.3 ± 0.3	Intervention group: 4.3 ± 0.4 Control group: 4.3 ± 0.4
History of cardiovascular disease, no.	Intervention group: 1,303 Control group: 1,302	Intervention group: 17 Control group: 3	Intervention group: 1,676 Control group: 1,654	NA	Intervention group: 242 Control group: 27	Intervention group: 45 Control group: 14	Intervention group: 23 Control group: 25	Intervention group: 47 Control group: 45
Heart failure, no.	Intervention group: 195 Control group: 241	NA	Intervention group: 290 Control group: 281	NA	NA	NA	Intervention group: 2 Control group: 1	Intervention group: 13 Control group: 12
Smoking history, no.	Intervention group: Never 1,375, former 1,044, current 414 Control group: Never 1,371, former 1,078, current 392	Intervention group: Never 22, former 38, current 24 Control group: Never 3, former 3, current 6	Intervention group: Never 1760, former 1,275, current 651 Control group: Never 1,684, former 1,346, current 636	NA	Intervention group: Never 280, former 309, current 138 Control group: Never 40, former 36, current 18	NA	NA	Intervention group: Never 142, former 819, current 41 Control group: Never 137, former 91, current 39
Baseline medications, no.	Intervention group: ACEI 950, ARB 1879, diuretic 1,577, GLP-1RA 189, SGLT2i 124 Control group: ACEI 992, ARB 1846, diuretic 1,637, GLP-1RA 205, SGLT2i 135	Intervention group: ACEI 9, ARB 75, diuretic 19 Control group: ACEI 0, ARB 12, diuretic 3	Intervention group: ACEI/ARB 3681, diuretic 1748, GLP-1RA 308, SGLT2i 314 Control group: ACEI/ARB 3662, diuretic 1748, GLP-1RA 242, SGLT2i 304	Intervention group: ACEI/ARB 141 Control group: ACEI/ARB 46	Intervention group: ACEI 334, ARB 397, diuretics 496 Control group: ACEI 41, ARB 55, diuretics 68	Intervention group: ACEI 8, ARB 277, diuretics 37, SGLT-2i 23 Control group: ACEI 2, ARB 71, diuretics 8, SGLT-2i 3	Intervention group: ACEI 13, ARB 209, diuretics 16, SGLT-2i 50, GLP-1RA 14 Control group: ACEI 13, ARB 214, diuretics 26, SGLT-2i 56, GLP-1RA 18	Intervention group: ACEI/ARB 40, diuretic 7, GLP-1RA 14, SGLT2i 32 Control group: ACEI/ARB 39, diuretic 7, GLP-1RA 18, SGLT2i 33

BMI, body mass index; SBP, systolic blood pressure; DBP, diastolic blood pressure; eGFR, estimated glomerular filtration rate; UACR, urine albumin-creatinine ratio; ACEI, angiotensin-converting enzyme inhibitor; ARB, angiotensin-receptor bloker; GLP-1RA, glucagon-like peptide-1, receptor agonist, SGLT-2i, sodium-glucose cotransporter 2 inhibitor; NA, not applicable.

### Quality assessment

All eight included studies (Bakris et al., 2020; Bakris et al., 2015; Ito et al., 2020; Ito et al., 2019; Katayama et al., 2017; Pitt et al., 2021; Tanaka et al., 2025; Wada et al., 2021) were assessed as having low risk of bias across all five domains of the Cochrane RoB 2 tool for each prespecified outcome (Supplementary Figures S1, S2).

### Effects on UACR

As presented in Figure 2, a pooled analysis of eight trials involving 14,512 patients (Bakris et al., 2020; Bakris et al., 2015; Ito et al., 2020; Ito et al., 2019; Katayama et al., 2017; Pitt et al., 2021; Tanaka et al., 2025; Wada et al., 2021) showed that nsMRAs significantly reduced UACR, with a mean decrease of 0.36 on the natural-log scale (95% CI -0.44 to −0.28, P < 0.00001), corresponding to an approximate 30% relative reduction. In addition, nsMRAs were associated with significantly greater odds of achieving UACR remission compared with control (OR 8.22, 95% CI 4.30 to 15.69, P < 0.00001; Supplementary Figure S3).

**FIGURE 2:**
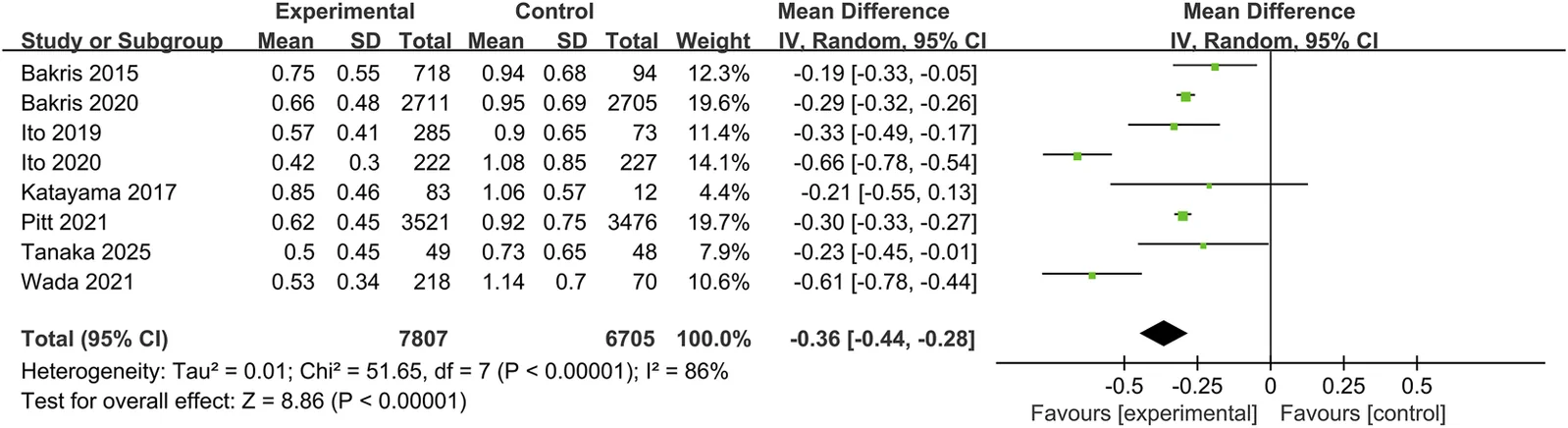
Forest plot of changes in log-transformed UACR between the nsMRAs and control groups.

### Effects on eGFR

A pooled analysis of three trials including 1,218 patients (Bakris et al., 2015; Ito et al., 2019; Tanaka et al., 2025) showed that nsMRAs therapy led to a greater decline in eGFR after 3-6 months of treatment relative to control (MD -3.01, 95% CI -4.46 to −1.56, P < 0.0001; Figure 3).

**FIGURE 3:**
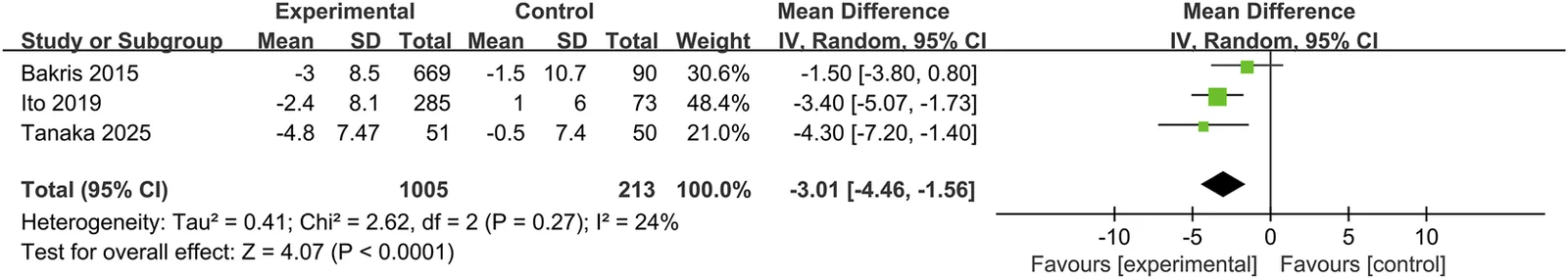
Forest plot of changes from baseline in eGFR between the nsMRAs and control groups.

### Effects on systolic BP

A pooled analysis of three trials involving 7,538 patients (Katayama et al., 2017; Pitt et al., 2021; Tanaka et al., 2025) showed that nsMRAs significantly reduced systolic BP compared with control (MD -4.72, 95% CI -8.64 to −0.80, P = 0.02; Figure 4). Additionally, three studies with missing SDs also demonstrated a consistent reduction, with nsMRAs lowering systolic BP by an average of 4.12 mmHg relative to control.

**FIGURE 4:**
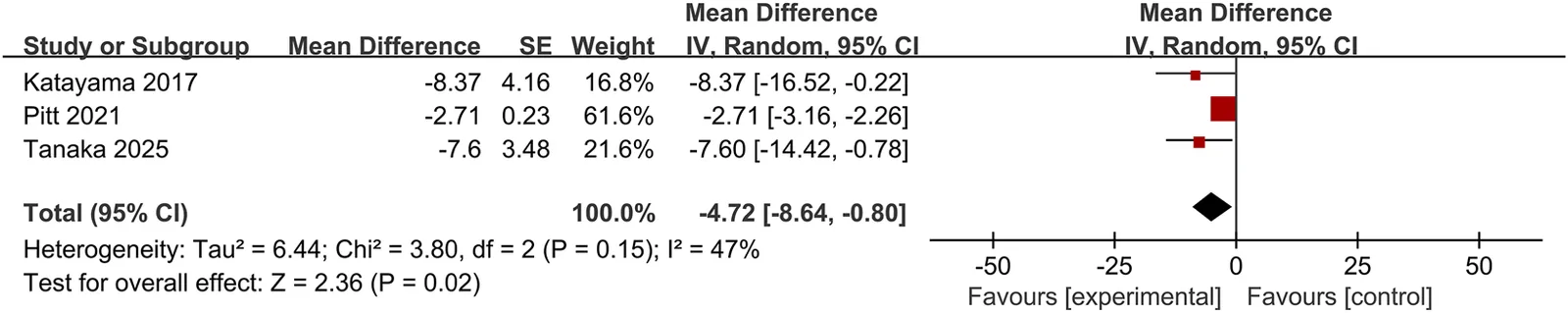
Forest plot of changes from baseline in systolic BP between the nsMRAs and control groups.

### Effects on serum potassium

As shown in Figure 5, a pooled analysis of four trials enrolling 1,468 patients (Bakris et al., 2015; Ito et al., 2019; Tanaka et al., 2025; Wada et al., 2021) showed that nsMRA therapy resulted in a greater increase in serum potassium versus control (MD 0.22, 95% CI 0.16 to 0.29, P < 0.00001). Similar to the pooled analysis, the two trials without SD data indicated an elevation in serum potassium, corresponding to a mean difference of 0.12 mmol/L for nsMRAs versus control.

**FIGURE 5:**
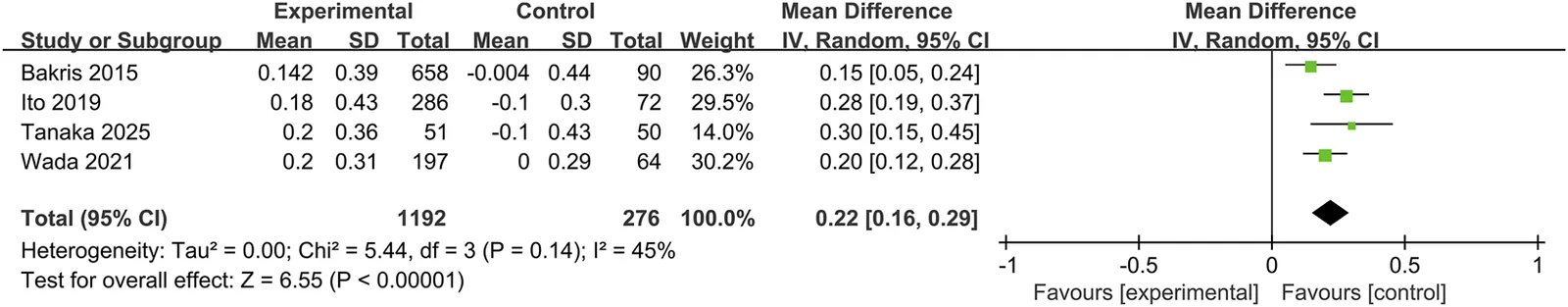
Forest plot of changes from baseline in serum potassium between the nsMRAs and control groups.

### Safety

Overall, the nsMRAs and control groups did not differ significantly in the total incidence of TEAEs (OR 0.99, 95% CI 0.90 to 1.09, P = 0.88; Table 2). When stratified by causality, the nsMRAs group exhibited higher rates of drug-related TEAEs (OR 1.57, 95% CI 1.33 to 1.84, P < 0.00001), treatment discontinuation due to TEAEs (OR 1.26, 95% CI 1.06 to 1.50, P = 0.007), and treatment discontinuation due to drug-related TEAEs (OR 7.09, 95% CI 1.88 to 26.70, P = 0.004). Drug-related serious TEAEs were numerically more common in the nsMRAs group (OR 1.37, 95% CI 0.99 to 1.89, P = 0.06), whereas treatment discontinuation due to serious TEAEs was comparable between groups (OR 0.95, 95% CI 0.75 to 1.19, P = 0.65). In contrast, the nsMRAs group had a significantly lower incidence of overall serious TEAEs (OR 0.91, 95% CI 0.85 to 0.98, P = 0.01).

**TABLE 2:** Comparison of TEAEs between the nsMRAs and control groups.

TEAEs	Studies	nsMRA group (*n/N*)	Control group (*n/N*)	OR (95% CI)	*P* Value (Effect)	Heterogeneity
**Any TEAEs**	7	6,395/7,884	5,885/6,946	0.99 (0.90, 1.09)	P = 0.88	P = 0.89, I^2^ = 0%
Treatment discontinuation due to TEAEs	7	498/8,020	366/7,006	1.26 (1.06, 1.50)	P = 0.009	P = 0.35, I^2^ = 10%
Drug-related TEAEs	6	1,307/7,157	888/6,852	1.57 (1.33, 1.84)	P < 0.00001	P = 0.19, I^2^ = 33%
Treatment discontinuation due to drug-related TEAEs	2	27/512	2/301	7.09 (1.88, 26.70)	P = 0.004	P = 0.95, I^2^ = 0%
**Any serious TEAEs**	6	2,129/7,800	2,228/6,934	0.91 (0.85, 0.98)	P = 0.01	P = 0.90, I^2^ = 0%
Treatment discontinuation due to serious TEAEs	3	162/7,237	155/6,583	0.95 (0.75, 1.19)	P = 0.65	P = 0.69, I^2^ = 0%
Drug-related serious TEAEs	5	96/7,574	62/6,705	1.37 (0.99, 1.89)	P = 0.06	P = 0.98, I^2^ = 0%
**Decline in eGFR** ^**&**^	5	206/4,150	138/3,103	1.08 (0.51, 2.26)	P = 0.84	P = 0.22, I^2^ = 31%
**AKI**	2	220/6,510	234/6,489	0.94 (0.78, 1.13)	P = 0.49	P = 0.88, I^2^ = 0%
AKI-related hospitalization	2	115/6,510	120/6,489	0.96 (0.74, 1.24)	P = 0.73	P = 0.77, I^2^ = 0%
Treatment discontinuation due to AKI	2	57/6,510	48/6,489	1.32 (0.54, 3.24)	P = 0.55	P = 0.03, I^2^ = 78%
**Hyperkalemia**	6	958/7,800	454/6,934	2.24 (1.99, 2.52)	P < 0.00001	P = 0.83, I^2^ = 0%
Serum potassium ≥ 5.5 mmol/L*	1	495/3,677	233/3,655	2.28 (1.94, 2.69)	P < 0.00001	NA
Serum potassium ≥ 6.0 mmol/L	3	119/4,189	49/3,956	2.33 (1.53, 3.53)	P < 0.00001	P = 0.34, I^2^ = 8%
Drug-related hyperkalemia	2	573/6,510	249/6,489	2.42 (1.97, 2.96)	P < 0.00001	P = 0.19, I^2^ = 43%
Serious hyperkalemia	2	69/6,510	16/6,489	4.27 (2.47, 7.38)	P < 0.00001	P = 0.41, I^2^ = 0%
Hyperkalemia-related hospitalization	2	61/6,510	10/6,489	5.92 (3.02, 11.62)	P < 0.00001	P = 0.38, I^2^ = 0%
Treatment discontinuation due to hyperkalemia	6	150/7,969	40/6,959	3.05 (2.14, 4.35)	P < 0.00001	P = 0.83, I^2^ = 0%
**Other common TEAEs**	​	​	​	​	​	​
Nasopharyngitis	4	656/7,523	591/6,655	0.98 (0.87, 1.10)	P = 0.70	P = 0.65, I^2^ = 0%
Upper respiratory tract infection	4	494/7,022	465/6,790	1.06 (0.92, 1.21)	P = 0.42	P = 0.58, I^2^ = 0%
Back pain	4	453/7,022	441/6,790	1.02 (0.89, 1.16)	P = 0.82	P = 0.91, I^2^ = 0%
Dizziness	4	365/7,523	324/6,655	1.06 (0.91, 1.24)	P = 0.44	P = 0.62, I^2^ = 0%
Constipation	4	324/6,820	341/6,730	0.93 (0.76, 1.15)	P = 0.51	P = 0.27, I^2^ = 23%
Anemia	3	426/6,594	397/6,501	1.07 (0.93, 1.23)	P = 0.34	P = 0.81, I^2^ = 0%
Diarrhea	3	446/7,237	413/6,583	1.03 (0.90, 1.19)	P = 0.67	P = 0.70, I^2^ = 0%
Hypokalemia	3	70/6,561	152/6,539	0.46 (0.34, 0.61)	P < 0.00001	P = 0.71, I^2^ = 15%
Blood uric acid increased	3	14/596	7/313	1.15 (0.45, 2.94)	P = 0.77	P = 0.82, I^2^ = 0%
Arthralgia	2	442/6,510	411/6,489	1.06 (0.89, 1.28)	P = 0.50	P = 0.21, I^2^ = 37%
Bronchitis	2	328/6,510	332/6,489	0.98 (0.81, 1.18)	P = 0.83	P = 0.24, I^2^ = 28%
Pneumonia	2	271/6,510	387/6,489	0.69 (0.58, 0.80)	P < 0.00001	P = 0.88, I^2^ = 0%
Peripheral edema	2	384/6,510	584/6,489	0.63 (0.54, 0.74)	P < 0.00001	P = 0.25, I^2^ = 25%
Hypertension	2	419/6,510	581/6,489	0.70 (0.60, 0.82)	P < 0.00001	P = 0.23, I^2^ = 30%
Urinary tract infection	2	431/6,510	432/6,489	0.99 (0.87, 1.14)	P = 0.93	P = 0.41, I^2^ = 0%
Hypoglycemia	2	340/6,510	375/6,489	0.89 (0.66, 1.20)	P = 0.46	P = 0.05, I^2^ = 74%
COVID-19^#^	2	85/3,734	116/3,708	0.72 (0.54, 0.96)	P = 0.02	P = 0.38, I^2^ = 0%
Serious COVID-19^#^	1	38/3,683	63/3,658	0.59 (0.40, 0.89)	P = 0.01	NA
Abdominal discomfort	2	10/512	12/301	0.53 (0.22, 1.26)	P = 0.15	P = 0.45, I^2^ = 0%
Others	5	110/1,374	44/457	0.98 (0.65, 1.48)	P = 0.91	P = 0.81, I^2^ = 0%

TEAEs, treatment-emergent adverse events; n, the number of patients who experienced TEAEs; N, the total number of patients included; eGFR, estimated glomerular filtration rate; AKI, acute kidney injury; NA, not applicable; OR, odds ratio; CI, confidence interval.

^&^ Decline in eGFR, was defined as a ≥ 30% decrease from baseline.

* Data for serum potassium ≥ 5.5 mmol/L were derived from a single trial (FIGARO-DKD). Because only one study contributed data, heterogeneity analysis was not possible, and the result should be interpreted with caution.

^#^ Data for COVID-19, were derived primarily from the FIDELIO-DKD, trial, with minimal contribution from the FIVE-STAR, trial; data for serious COVID-19, were derived solely from the FIDELIO-DKD, trial. These analyses were exploratory and *post hoc*, lacked multiplicity correction, and were susceptible to confounding by geography and pandemic timing. Therefore, the results should be interpreted with caution and should not be extrapolated to the broader nsMRA, class.

Renal safety assessments showed no significant between-group differences in the overall rate of eGFR decline (OR 1.08, 95% CI 0.51 to 2.26, P = 0.84), AKI (OR 0.94, 95% CI 0.78 to 1.13, P = 0.49), AKI-related hospitalization (OR 0.96, 95% CI 0.78 to 1.13, P = 0.73), or treatment discontinuation due to AKI (OR 1.32, 95% CI 0.54 to 3.24, P = 0.55).

However, hyperkalemia was consistently more common with nsMRA therapy (OR 2.24, 95% CI 1.99 to 2.52, P < 0.00001). The excess risk spanned both biochemical and clinical endpoints: serum potassium ≥ 5.5 mmol/L (OR 2.28, 95% CI 1.94 to 2.69, based on a single study) and ≥ 6.0 mmol/L (OR 2.33, 95% CI 1.53 to 3.53, P < 0.00001); drug-related hyperkalemia (OR 2.42, 95% CI 1.97 to 2.96, P < 0.00001); serious hyperkalemia (OR 4.27, 95% CI 2.47 to 7.38, P < 0.00001), hyperkalemia-related hospitalization (OR 5.92, 95% CI 3.02 to 11.62, P < 0.00001), and treatment discontinuation due to hyperkalemia (OR 3.05, 95% CI 2.14 to 4.35, P < 0.00001).

For other frequently reported TEAEs, nsMRAs therapy was associated with lower risks of hypokalemia (OR 0.46, 95% CI 0.34 to 0.61, P < 0.00001), peripheral edema (OR 0.63, 95% CI 0.54 to 0.74, P < 0.00001), hypertension (OR 0.70, 95% CI 0.60 to 0.82, P < 0.00001), and pneumonia (OR 0.69, 95% CI 0.58 to 0.80, P < 0.00001).

### Sensitivity analysis

For changes in serum potassium from baseline, SDs were unavailable in two trials (Bakris et al., 2020; Pitt et al., 2021). We therefore estimated them using a correlation-based imputation approach. As shown in Supplementary Figure S4, the imputed SDs for the nsMRA group were 0.45 (Pitt et al., 2021) and 0.48 (Bakris et al., 2020), both slightly exceeding the SD range (0.36-0.43) among studies contributing to the imputation. Similarly, the imputed SDs for the control group were 0.47 (Pitt et al., 2021) and 0.51 (Bakris et al., 2020), also slightly above the corresponding SD range of the contributing studies (0.30-0.43). The pooled MD for serum potassium was 0.17 (95% CI 0.12 to 0.21, P < 0.00001) when using imputed SDs, which was consistent with the results excluding these imputed studies (MD 0.22, 95% CI 0.16 to 0.29). However, heterogeneity (I^2^) increased markedly from 45% to 78% after imputation. Furthermore, varying the assumed correlation coefficient by ±0.2 in a one-way sensitivity analysis did not materially alter the direction or statistical significance of the pooled estimates. Nonetheless, significant heterogeneity (I^2^) persisted in both sensitivity analyses (77% for a correlation coefficient of −0.2 and 79% for +0.2; Supplementary Figure S5, S6). Given the substantial and persistent increase in heterogeneity introduced by the imputed values, the primary analysis for serum potassium was therefore based on the non-imputed data.

Robustness of the pooled estimates was evaluated via leave-one-out analyses, wherein each study was sequentially omitted. For change in UACR (Supplementary Figure S7), change in serum potassium (Supplementary Figure S8), and change in eGFR (Supplementary Figure S9), the meta-analytic effect sizes proved robust across all iterations, with no individual study identified as an outlier driving the results. However, for change in systolic BP, although the direction and statistical significance of the pooled estimate remained unchanged, we observed that the FIGARO-DKD trial (Pitt et al., 2021) exerted a disproportionately large influence on the pooled estimate (Supplementary Figure S10).

Additionally, to assess the impact of short-term studies (follow-up duration less than 24 weeks), we performed a sensitivity analysis excluding these short-term trials. Except for eGFR, the direction and statistical significance of the effects of nsMRAs on all primary efficacy outcomes consistent with the primary analysis: UACR (MD - 0.41, 95% CI -0.51 to −0.31, P < 0.00001; Supplementary Figure S11), systolic BP (MD -3.92, 95% CI -8.06 to 0.22; Supplementary Figure S13), and serum potassium (MD 0.23, 95% CI 0.14 to 0.32, P < 0.00001; Supplementary Figure S14). For eGFR, the direction remained consistent, but statistical significance was lost (MD -4.30, 95% CI -7.20 to −1.40; Supplementary Figure S12), changing from significant in the primary analysis (P = 0.02) to non-significant in the sensitivity analysis (P = 0.06), likely reflecting reduced statistical power after excluding short-term trials. For the prespecified aggregate safety outcomes (any TEAE, any serious TEAEs, and TEAE leading to discontinuation), the results were consistent with the primary analysis (Supplementary Table S3). These findings indicate that the inclusion of short-term trials did not materially affect the direction of the primary efficacy or prespecified safety outcomes, although the loss of statistical significance for eGFR should be interpreted with caution.

### Subgroup analysis

Exploratory subgroup analyses showed that nsMRAs consistently reduced UACR levels. Subgroup analyses stratified by sample size (Supplementary Figure S15), age (Supplementary Figure S16), study duration (Supplementary Figure S17), baseline UACR (Supplementary Figure S18), and baseline eGFR (Supplementary Figure S19) revealed no statistically significant between-subgroup interactions. One study (Katayama et al., 2017) was excluded from the age-based subgroup analysis due to significant baseline age imbalance between the intervention and control arms. Notably, although sample size and baseline UACR are distinct stratification variables, they yielded identical subgroup allocations in our dataset because of the specific distribution of these characteristics across the included trials (trial characteristics and subgroup membership are summarized in Supplementary Table S4). This overlap implies that the two analyses actually examined the same between-subgroup contrasts. In contrast, stratification by drug type (Supplementary Figure S20) yielded statistically significant between-subgroup interactions. While nsMRAs consistently reduced UACR within each drug-specific subgroup, these findings suggest that drug type may be a potential source of inter-study heterogeneity.

Regarding serum potassium, nsMRAs consistently elevated its levels across studies. Subgroup analyses based on drug type (Supplementary Figure S21), sample size (Supplementary Figure S22), and study duration (Supplementary Figure S23) showed no significant interactions, with sample size and study duration partitioning the same trials into complementary subgroups (trial characteristics and subgroup membership are summarized in Supplementary Table S5). However, stratification by age revealed a statistically significant between-subgroup interaction (Supplementary Figure S24). Although nsMRAs increased serum potassium levels within all age subgroups, this result suggests that age may contribute to heterogeneity across studies.

For systolic BP and eGFR decline, subgroup analyses were not performed, as each of these outcomes comprised only three studies, precluding any meaningful subgroup exploration.

### Certainty of evidence

As shown in Supplementary Table S6, the certainty of evidence was rated as low for UACR, eGFR, systolic BP, and serum potassium, and moderate for hyperkalemia. For UACR, the evidence was downgraded by one level for substantial heterogeneity and by an additional level for imprecision, as the 95% CI on the log scale (−0.44 to −0.28) crossed the MID of −0.36. For eGFR, the evidence was downgraded by one level for inconsistency due to potential methodological differences in calculation formulas across studies. Regarding imprecision, the median control-group baseline SD across the three contributing studies was 7.4 mL/min/1.73 m^2^ (SDs: 6.0, 7.4, and 10.7), yielding a calculated MID of 3.7 mL/min/1.73 m^2^ (0.5 × 7.4). Because the 95% CI (−4.46 to −1.56) crossed this MID, imprecision was downgraded by one level. This was further supported by the sensitivity analysis excluding short-term trials, in which statistical significance was lost although the direction remained consistent. Therefore, eGFR was rated as low certainty of evidence. For systolic BP, three of six studies did not report SDs, precluding their inclusion in the meta-analysis. Consequently, we downgraded one level for risk of bias. Moreover, the pooled estimate from the remaining studies was heavily driven by a single study, which prompted a further downgrade of one level for imprecision. For serum potassium, we downgraded one level for risk of bias, as two large studies were excluded after imputation yielded implausibly high SDs. Regarding imprecision, the median control-group baseline SD across the four contributing studies was 0.365 mmol/L (SDs: 0.29, 0.30, 0.43, and 0.44), yielding a calculated MID of 0.18 mmol/L (0.5 × 0.365). The 95% CI (0.16 to 0.29) crossed this calculated MID, prompting a further downgrade of one level for imprecision. For hyperkalemia, the evidence was downgraded by one level for inconsistency, owing to variations in outcome definitions across trials.

## Discussion

In this updated meta-analysis, we addressed key limitations of the previous meta-analysis (Jiang et al., 2022) on the efficacy of nsMRAs in patients with DKD. Specifically, we incorporated all available dose data (including recently published trials), systematically evaluated safety outcomes, and applied the GRADE framework to assess the certainty of evidence. These methodological enhancements enable a more comprehensive and reliable evaluation of both efficacy and safety of nsMRAs in this population. Our findings suggest that nsMRAs may be associated with reductions in UACR and systolic BP, although the certainty of these findings was low. Moreover, our meta-analysis showed that nsMRAs may be associated with an early decline in eGFR, which likely reflects a hemodynamic adaptation rather than structural renal injury. On the safety side, nsMRAs were associated with an increase in serum potassium and an elevated risk of hyperkalemia-related events, underscoring the need for vigilant monitoring. Notably, our review assessed only surrogate endpoints (UACR, eGFR, systolic BP, and UACR remission rate) and safety outcomes, and did not evaluate hard cardiorenal endpoints (e.g., ESRD, cardiovascular death, or heart failure hospitalization). Thus, our findings suggest potential cardiorenal benefits but cannot confirm hard endpoint benefits. Although the original large-scale trials (FIDELIO-DKD and FIGARO-DKD) included in this review reported hard endpoint benefits, these endpoints were not analyzed in our meta-analysis and therefore should not be attributed to the present review. Finally, given these uncertainties, clinical use of nsMRAs requires careful risk-benefit assessment.

Within the DKD population, albuminuria serves as both a diagnostic cornerstone and a robust prognostic marker for disease progression (Lin et al., 2018). Given its prognostic value, changes in albuminuria have been adopted as a surrogate endpoint for cardiorenal outcomes in this population (Palmer, 2023). In our meta-analysis, nsMRAs yielded an approximately 30% reduction in UACR and significantly improved the UACR remission rate. The leave-one-out sensitivity analysis showed that this pooled estimate was not dominated by any individual study, suggesting statistical robustness. However, this robustness was observed within an evidence base that was predominantly driven by finerenone, which contributed 5 of 8 included trials. Consistent with this, a large-scale meta-analysis (Chen et al., 2024) demonstrated a similar 30% UACR reduction with finerenone across 13,943 DKD patients. In contrast, another pooled analysis of five clinical trials (Uchida et al., 2025) observed a 56.5% UACR reduction after 12 weeks of esaxerenone therapy in patients with type 2 diabetes and CKD.

Nevertheless, significant heterogeneity in the effect of nsMRAs on UACR was observed in our meta-analysis. Exploratory subgroup analyses showed a statistically significant drug-type interaction, suggesting that drug type might be a major source of this variation, with esaxerenone appearing to confer a greater reduction in UACR than finerenone or apararenone. This result is consistent with the findings of the previous meta-analysis (Jiang et al., 2022). Given that finerenone dominates the evidence base, whereas esaxerenone and apararenone contribute comparatively little data, the UACR findings should be interpreted at the agent-specific level where data are available, rather than as a uniform class effect. In addition, according to the GRADE approach, the certainty of evidence for nsMRAs in reducing UACR was assessed as low. Therefore, given the substantial heterogeneity, the significant drug-type interaction, and the low certainty of evidence, the conclusions regarding UACR reduction should be interpreted with caution.

Currently, the exact pathophysiological basis for the antiproteinuric effect of nsMRAs remains to be fully elucidated. However, mechanistic insights can be drawn from a previous study (Okuda et al., 2023), which quantified the relative contributions of hemodynamic factors. In that analysis, changes in eGFR emerged as the dominant mediator, explaining approximately 24.3% of the UACR reduction, while systolic BP lowering accounted for roughly 9.8%. These findings suggest that improved glomerular hemodynamics may be a key mechanism underlying the UACR response to esaxerenone, whereas the antihypertensive effect appears to play a secondary role. Beyond hemodynamics, molecular mechanisms may also contribute to renoprotection, including the endothelial glycocalyx, modulation of matrix metalloproteinases, and Rac1-dependent stabilization of the podocyte cytoskeleton (Crompton et al., 2023; Ebefors et al., 2019; Gil-Ortega et al., 2020; Shibata et al., 2008). However, whether these hemodynamic and molecular mechanisms apply equally to other nsMRAs remains uncertain.

As a cornerstone surrogate marker for renal function, the trajectory of eGFR decline has traditionally served as a robust prognostic indicator of adverse renal outcomes in individuals with DKD (Cao et al., 2025; Lambers Heerspink et al., 2014; Shimizu et al., 2018). Contemporary evidence, however, suggests a revised interpretation of early on-treatment eGFR reductions, which are now reframed as a reflection of glomerular hemodynamic modulation rather than parenchymal damage (Agarwal et al., 2022; Bakris and Weir, 2022). In line with this, our meta-analysis suggested that nsMRAs were associated with a more pronounced eGFR reduction within the initial 3-6 months of therapy. This finding was robust in the leave-one-out sensitivity analysis, although it was based on only three studies. However, in the sensitivity analysis excluding short-term trials, the statistical significance of the eGFR change was lost, despite a consistent direction. This attenuation likely reflects reduced statistical power, but it also indicates that the early eGFR-reducing effect was not entirely robust. Nevertheless, safety profiling provided indirect support for a hemodynamic rather than a structural basis: there was no concomitant elevation in sustained eGFR loss, AKI, or AKI-related events. If the eGFR decline had been due to structural damage, an increase in AKI events would have been expected. Since this was not observed, a hemodynamic mechanism appears more plausible. However, it is important to emphasize that this hemodynamic interpretation remains a hypothesis rather than a conclusion directly demonstrated by the present meta-analysis, as our pooled dataset did not include serial eGFR trajectories or assess reversibility at the individual level. Nonetheless, consistent with this hypothesis, trial-level data from individual studies have described a characteristic early eGFR decline followed by recovery. For instance, a randomized placebo-controlled study (Ito et al., 2019) reported that esaxerenone induced a modest eGFR reduction as early as week 4, reaching its nadir by week 8, with gradual recovery thereafter in DKD patients. Similarly, the CONFIDENCE trial (Agarwal et al., 2025) showed that finerenone triggered a rapid, mild eGFR decline from day 14, bottoming out at day 90, followed by a subsequent rebound. Moreover, the observed nadir-and-rebound trajectory at the group level raises the hypothesis that the early dip might translate into the durable renal benefits reported over the long term, as observed in the FIDELIO-DKD and FIGARO-DKD trials (Bakris et al., 2020; Pitt et al., 2021), indirectly supporting the clinical relevance of this hemodynamic phenomenon. Given this characteristic pattern, serial renal function assessments during the early phase of nsMRA therapy may be clinically prudent to differentiate hemodynamic adaptive changes from structural kidney injury. Nevertheless, it should be noted that the certainty of evidence for the early eGFR-reducing effect of nsMRAs was rated as low in our meta-analysis, owing to inconsistency and imprecision. Therefore, the findings warrant particularly cautious interpretation.

Inadequate BP management is closely correlated with the progression of adverse cardiorenal events in DKD patients (Burnier and Damianaki, 2023; Ku et al., 2019; Van Buren and Toto, 2011). Accordingly, intensified antihypertensive therapy is expected to confer dual benefits on renal and cardiovascular outcomes. Our meta-analysis showed that nsMRAs significantly reduced systolic BP in DKD patients, a finding that was robust in the leave-one-out sensitivity analysis, albeit limited to only three studies. Moreover, safety analysis revealed a significantly lower incidence of hypertension in the nsMRAs group than in the control group. These results were further supported by three additional studies not included in the pooled analysis, which consistently reported a systolic BP-lowering effect. These results are in line with the observations of a real-world study (Wu et al., 2025), which found that finerenone significantly reduced systolic BP in patients with CKD and type 2 diabetes after 6 months of treatment. Another pooled analysis of five clinical studies (Uchida et al., 2025) also noted a sustained antihypertensive effect of esaxerenone in CKD patients, regardless of diabetes status. More importantly, esaxerenone has been approved in Japan for the treatment of hypertension (Duggan, 2019). Accordingly, this evidence suggests that nsMRAs may confer a clinically meaningful reduction in systolic BP among DKD patients.

However, several limitations weaken this conclusion. One large-scale study (Pitt et al., 2021) contributed a disproportionately large weight to the pooled analysis, which may have biased the overall estimate. In addition, three eligible trials were excluded from the quantitative synthesis, which may have affected the precision of our estimates. Moreover, the GRADE certainty of evidence for nsMRAs in lowering systolic BP in the primary analysis was rated as low. Therefore, the aforementioned systolic BP findings in our study should be considered preliminary and exploratory, warranting further validation in larger and more homogeneous cohorts.

Beyond therapeutic efficacy, we systematically evaluated the safety profile of nsMRAs in DKD patients. Relative to control, nsMRA therapy was associated with a higher incidence of drug-related TEAEs and TEAE-related treatment discontinuation, whereas the incidence of total TEAEs was comparable and the risks of serious TEAEs and TEAE-associated death were lower. These findings suggest that, despite an increased risk of drug-related adverse events, nsMRAs exhibit a generally favorable and clinically manageable safety profile.

Notably, steroidal MRAs have a well-recognized risk of hyperkalemia. The magnitude of this risk with nsMRAs appears to be lower. However, it remains a clinically relevant concern in the DKD setting. Pooled estimates from our analysis showed that nsMRA therapy produced a statistically significant rise in serum potassium relative to control. The leave-one-out sensitivity analysis showed that this finding was robust and not driven by any single study. Safety profiling further revealed a heightened risk for potassium-related adverse events, including hyperkalemia, serious hyperkalemia, hospitalization due to hyperkalemia, and treatment discontinuation due to hyperkalemia. These observations are in line with prior meta-analyses (Bao et al., 2022; Yasmin et al., 2023), both of which reported hyperkalemic signals associated with finerenone across diverse DKD populations. However, a major limitation must be emphasized: the two largest and most influential nsMRA trials, FIDELIO-DKD (Bakris et al., 2020) and FIGARO-DKD (Pitt et al., 2021), were excluded from the primary pooled analysis of serum potassium because they did not report the corresponding SDs.

Although we attempted to impute the missing SDs using the correlation coefficient method, the imputed results showed a marked increase in heterogeneity, suggesting that the imputed values may introduce substantial uncertainty into the pooled estimates. In addition, although the primary analysis of serum potassium did not reveal significant heterogeneity, exploratory subgroup analyses suggested that age might be a potential source of heterogeneity. It is well recognized that age is closely related to baseline eGFR, likely through its association with renal function, and baseline eGFR may modulate the serum potassium response to nsMRAs. However, due to data limitations, we were unable to perform subgroup analyses by baseline eGFR and could not assess its modifying effect. Moreover, the GRADE certainty of evidence was rated as low for nsMRAs increasing serum potassium and moderate for causing hyperkalemia. Given that hyperkalemia is arguably the most clinically important safety signal for this drug class, the exclusion of the FIDELIO-DKD and FIGARO-DKD trials (Bakris et al., 2020; Pitt et al., 2021) substantially limits the precision and generalizability of our serum potassium estimate. Taken together, these limitations warrant particularly cautious interpretation of the serum potassium results.

Additionally, despite the risk of hyperkalemia, our analysis revealed a significant reduction in hypokalemia risk. This observation may be attributable to the potassium-retaining effect of MRAs. Notably, although this counterbalancing effect may help maintain normokalemia, hyperkalemia remains the clinically dominant concern of nsMRAs.

Owing to data constraints, our meta-analysis did not evaluate new-onset hyperkalemia risk. Nevertheless, evidence indicates that a constellation of baseline covariates independently predicts incident hyperkalemia in DKD patients receiving finerenone, namely,: prior hyperkalemia, serum potassium > 4.5 mmol/L, UACR > 1,000 mg/g, eGFR < 45 mL/min/1.73 m^2^, hemoglobin < 120 g/L, and lack of concomitant SGLT2i or thiazide diuretic therapy (Ferreira et al., 2025). Accordingly, the clinical use of nsMRAs mandates vigilant potassium monitoring, with particular attention to patients presenting with multiple concurrent risk factors.

Currently, RAASi and SGLT2i are considered the foundational therapy for DKD management (Kidney Disease: Improving Global Outcomes Diabetes Work Group, 2022). However, in clinical practice, the optimization of RAASi and SGLT2i does not eliminate residual cardiorenal risk in all DKD patients, often necessitating a tailored approach to subsequent therapy. The latest KDIGO guidelines support a phenotype-guided selection between GLP-1RAs and finerenone, a representative agent of nsMRAs (Kidney Disease: Improving Global Outcomes Diabetes Work Group, 2022). Specifically, the presence of high-volume proteinuria, rapid renal function decline, or concomitant heart failure generally favors nsMRAs. These agents attenuate MR-associated fibrotic cascades and address tissue remodeling that may persist despite SGLT2i use. Conversely, GLP-1RAs are typically preferred for patients with predominantly macrovascular atherosclerosis with stable renal parameters. They offer comprehensive cardiometabolic protection through GLP-1R engagement, a pathway distinct from MR signaling. For complex cases exhibiting overlapping phenotypes, a four-drug strategy may be considered. However, short-term renal function trajectories and serum potassium elevation should be monitored.

The biological rationale for such combinations lies in their complementary actions across multiple pathways. SGLT2i induces a transient, early-phase dip in eGFR via preglomerular arteriolar vasoconstriction (Husain-Syed et al., 2026; Provenzano et al., 2021). This reflects effective glomerular decongestion. RAASi primarily modulates efferent arteriolar tone to reduce intraglomerular pressure (Husain-Syed et al., 2026; Lewis, 2002). nsMRAs complement these hemodynamic effects by suppressing MR-driven inflammation and fibrosis, thereby mitigating aldosterone-mediated tissue injury (Husain-Syed et al., 2026; Vodošek Hojs et al., 2021). Beyond this complementary interplay, nsMRAs and GLP-1RAs engage distinct and non-overlapping disease mechanisms. nsMRAs block MR-mediated organ fibrosis, whereas GLP-1RAs curb atherosclerotic and metabolic risk by activating GLP-1R. This enables concurrent targeting of fibrotic and atherosclerotic pathways in patients with mixed phenotypes. From a safety standpoint, both RAASi and nsMRAs carry a risk of hyperkalemia. SGLT2i promotes urinary potassium loss, creating a physiological buffer against such hyperkalemia risk and broadening the therapeutic window for combination regimens.

### Limitations

Several limitations of this meta-analysis should be acknowledged. First, the included studies focused predominantly on finerenone, whereas data on other nsMRA agents (e.g., esaxerenone and apararenone) were sparse. In addition, hard cardiorenal endpoints were reported only in the two large, long-term finerenone trials (FIDELIO-DKD and FIGARO-DKD). Other nsMRAs were evaluated in shorter-term trials without hard endpoint data. Therefore, hard endpoint benefits observed with finerenone cannot be directly extrapolated to other nsMRAs, and our review was limited to surrogate endpoints and safety outcomes. This uneven distribution of evidence limits the generalizability of our findings. Moreover, the drug-type subgroup interaction for UACR was statistically significant, suggesting that the UACR-lowering effect may differ across nsMRA agents. Because finerenone dominates the evidence base while esaxerenone and apararenone contribute comparatively little data, class-level generalization of the UACR findings should be made with caution, and the results should be interpreted at the agent-specific level where data are available. Second, considerable heterogeneity across studies in patient populations, treatment durations, background therapies, and the timing of outcome assessments may have influenced the observed effect estimates. This was particularly evident for UACR, as reflected by the significant drug-type interaction described in the first limitation. This heterogeneity also affected the certainty of evidence: variations in outcome definitions contributed to moderate certainty for hyperkalemia, whereas methodological discrepancies in calculation formulas and imprecision contributed to low certainty for eGFR. Third, the limited number of included studies precluded formal meta-regression and publication bias assessment, limiting our ability to explore sources of heterogeneity or detect potential reporting bias. Fourth, data availability for certain continuous outcomes posed additional analytical challenges. For systolic BP, three of the six studies lacked SD values, precluding reliable imputation. Therefore, the pooled estimate was based on the remaining three trials. Notably, this estimate was driven predominantly by the FIGARO-DKD trial, limiting the generalizability of the pooled result. Similarly, for serum potassium, two of the six studies had missing SDs, namely, the two largest trials (FIDELIO-DKD and FIGARO-DKD). Although imputation was attempted for these missing SDs, it substantially increased statistical heterogeneity. Consequently, the imputed values were not included in the primary analysis. While this approach avoided the increase in heterogeneity, it may have reduced the precision of the pooled estimates. Fifth, in the subgroup analyses, subgroup definitions were not identical across outcomes because the number of studies contributing to each outcome differed substantially. This limits direct comparisons of subgroup findings across outcomes, and all subgroup results should therefore be considered exploratory and hypothesis-generating. Sixth, analyses of individual TEAEs were exploratory, and no multiplicity adjustment was applied. P values for individual TEAEs should therefore be interpreted cautiously because of the potential for Type I error. In addition, because the original studies only reported adverse events above certain frequency thresholds, selective reporting of TEAEs cannot be fully excluded. Seventh, the original PROSPERO protocol specified a minimum follow-up of 24 weeks, but this criterion was removed via a major revision to include three short-term trials (3-month follow-up). A sensitivity analysis excluding these short-term trials showed that the effects of nsMRAs on UACR, systolic BP, serum potassium, and the prespecified aggregate safety outcomes were consistent with the primary analysis; however, for change in eGFR, statistical significance was lost, although the direction remained consistent. Therefore, the inclusion of shorter-term studies may limit the robustness of the early eGFR-reducing effect and the assessment of long-term renal outcomes. Collectively, these methodological and data-related constraints warrant cautious interpretation of the pooled estimates, which should be regarded as exploratory rather than definitive.

## Conclusion

In patients with DKD, nsMRAs may reduce UACR and systolic BP. However, the UACR estimate was predominantly driven by finerenone (5 of 8 trials), and a significant drug-type interaction was observed; therefore, UACR findings should be interpreted at the agent-specific level rather than as a uniform class effect. The systolic BP estimate was also based on limited and heterogeneous data and was dominated by a single large trial (FIGARO-DKD). In addition, nsMRAs may be associated with an early decline in eGFR, which likely reflects a hemodynamic adaptation rather than true functional decline. Moreover, nsMRAs were associated with an increase in serum potassium and an elevated risk of hyperkalemia, although the serum potassium analysis excluded the two largest trials (FIDELIO-DKD and FIGARO-DKD). Therefore, routine monitoring of serum potassium and eGFR is warranted before and during therapy, particularly in patients with preexisting renal impairment or a predisposition to hyperkalemia. Notably, this review assessed only surrogate endpoints and safety outcomes, not hard cardiorenal endpoints. Furthermore, several outcomes were supported by only 3-4 trials. Given the low to moderate certainty of evidence and these limitations, these findings should be interpreted with caution, and the risk-benefit balance should be carefully considered in individual patients.

## Data Availability

The original contributions presented in the study are included in the article/Supplementary Material, further inquiries can be directed to the corresponding authors.
