# Supplementary material for: Efficacy and safety of novel non-steroidal mineralocorticoid receptor antagonists in diabetic kidney disease: an updated systematic review and meta-analysis of randomized controlled trials
Source: Front Pharmacol. 2026 Oct 28;17:1983608. doi: 10.3389/fphar.2026.1983608 (PMC13752123; doi:10.3389/fphar.2026.1983608)
Supplement: Supplementary file 1 [file DataSheet1.pdf]

# Supplementary Materials

**Table S1**. PRISMA 2020 checklist.

| Section and Topic | Item # | Checklist item | Location where item is reported |
|---|---|---|---|
| **TITLE** | | | |
| Title | 1 | Identify the report as a systematic review. | Page 1 |
| **ABSTRACT** | | | |
| Abstract | 2 | See the PRISMA 2020 for Abstracts checklist. | Page 1-2 |
| **INTRODUCTION** | | | |
| Rationale | 3 | Describe the rationale for the review in the context of existing knowledge. | Page 2-3 |
| Objectives | 4 | Provide an explicit statement of the objective(s) or question(s) the review addresses. | Page 3 |
| **METHODS** | | | |
| Eligibility criteria | 5 | Specify the inclusion and exclusion criteria for the review and how studies were grouped for the syntheses. | Page 3 |
| Information sources | 6 | Specify all databases, registers, websites, organisations, reference lists and other sources searched or consulted to identify studies. Specify the date when each source was last searched or consulted. | Page 3 |
| Search strategy | 7 | Present the full search strategies for all databases, registers and websites, including any filters and limits used. | Page 3 |
| Selection process | 8 | Specify the methods used to decide whether a study met the inclusion criteria of the review, including how many reviewers screened each record and each report retrieved, whether they worked independently, and if applicable, details of automation tools used in the process. | Page 4 |
| Data collection | 9 | Specify the methods used to collect data from reports, including how many reviewers collected data from each report, whether | Page 4 |

| Section and Topic | Item # | Checklist item | Location where item is reported |
|---|---|---|---|
| process | | they worked independently, any processes for obtaining or confirming data from study investigators, and if applicable, details of automation tools used in the process. | |
| Data items | 10a | List and define all outcomes for which data were sought. Specify whether all results that were compatible with each outcome domain in each study were sought (e.g. for all measures, time points, analyses), and if not, the methods used to decide which results to collect. | Page 4-5 |
| | 10b | List and define all other variables for which data were sought (e.g. participant and intervention characteristics, funding sources). Describe any assumptions made about any missing or unclear information. | Page 4-5 |
| Study risk of bias assessment | 11 | Specify the methods used to assess risk of bias in the included studies, including details of the tool(s) used, how many reviewers assessed each study and whether they worked independently, and if applicable, details of automation tools used in the process. | Page 3-4 |
| Effect measures | 12 | Specify for each outcome the effect measure(s) (e.g. risk ratio, mean difference) used in the synthesis or presentation of results. | Page 4 |
| Synthesis methods | 13a | Describe the processes used to decide which studies were eligible for each synthesis (e.g. tabulating the study intervention characteristics and comparing against the planned groups for each synthesis (item #5)). | Page 5-7 |
| | 13b | Describe any methods required to prepare the data for presentation or synthesis, such as handling of missing summary statistics, or data conversions. | Page 5-7 |
| | 13c | Describe any methods used to tabulate or visually display results of individual studies and syntheses. | Page 5-7 |
| | 13d | Describe any methods used to synthesize results and provide a rationale for the choice(s). If meta-analysis was performed, describe the model(s), method(s) to identify the presence and extent of statistical heterogeneity, and software package(s) used. | Page 5-7 |
| | 13e | Describe any methods used to explore possible causes of heterogeneity among study results (e.g. subgroup analysis, meta-regression). | Page 5-7 |

| Section and Topic | Item # | Checklist item | Location where item is reported |
|---|---|---|---|
| | 13f | Describe any sensitivity analyses conducted to assess robustness of the synthesized results. | Page 5-7 |
| Reporting bias assessment | 14 | Describe any methods used to assess risk of bias due to missing results in a synthesis (arising from reporting biases). | Page 7 |
| Certainty assessment | 15 | Describe any methods used to assess certainty (or confidence) in the body of evidence for an outcome. | Page 7-8 |
| **RESULTS** | | | |
| Study selection | 16a | Describe the results of the search and selection process, from the number of records identified in the search to the number of studies included in the review, ideally using a flow diagram. | Page 8 |
| | 16b | Cite studies that might appear to meet the inclusion criteria, but which were excluded, and explain why they were excluded. | Page 8 |
| Study characteristics | 17 | Cite each included study and present its characteristics. | Page 8 |
| Risk of bias in studies | 18 | Present assessments of risk of bias for each included study. | Page 8 |
| Results of individual studies | 19 | For all outcomes, present, for each study: (a) summary statistics for each group (where appropriate) and (b) an effect estimate and its precision (e.g. confidence/credible interval), ideally using structured tables or plots. | Page 8 |
| Results of syntheses | 20a | For each synthesis, briefly summarise the characteristics and risk of bias among contributing studies. | Page 8-11 |
| | 20b | Present results of all statistical syntheses conducted. If meta-analysis was done, present for each the summary estimate and its precision (e.g. confidence/credible interval) and measures of statistical heterogeneity. If comparing groups, describe the direction of the effect. | Page 8-11 |

| Section and Topic | Item # | Checklist item | Location where item is reported |
|---|---|---|---|
| | 20c | Present results of all investigations of possible causes of heterogeneity among study results. | Page 10-11 |
| | 20d | Present results of all sensitivity analyses conducted to assess the robustness of the synthesized results. | Page 9-10 |
| Reporting biases | 21 | Present assessments of risk of bias due to missing results (arising from reporting biases) for each synthesis assessed. | Page 7 |
| Certainty of evidence | 22 | Present assessments of certainty (or confidence) in the body of evidence for each outcome assessed. | Page 11 |
| **DISCUSSION** | | | |
| Discussion | 23a | Provide a general interpretation of the results in the context of other evidence. | Page 11-16 |
| | 23b | Discuss any limitations of the evidence included in the review. | Page 11-16 |
| | 23c | Discuss any limitations of the review processes used. | Page 11-16 |
| | 23d | Discuss implications of the results for practice, policy, and future research. | Page 11-16 |
| **OTHER INFORMATION** | | | |
| Registration and protocol | 24a | Provide registration information for the review, including register name and registration number, or state that the review was not registered. | Page 3 |
| | 24b | Indicate where the review protocol can be accessed, or state that a protocol was not prepared. | Page 3 |
| | 24c | Describe and explain any amendments to information provided at registration or in the protocol. | Page 3 |
| Support | 25 | Describe sources of financial or non-financial support for the review, and the role of the funders or sponsors in the review. | Page 17 |
| Competing interests | 26 | Declare any competing interests of review authors. | Page 17-18 |

| Section and Topic | Item # | Checklist item | Location where item is reported |
|---|---|---|---|
| Availability of data, code and other materials | 27 | Report which of the following are publicly available and where they can be found: template data collection forms; data extracted from included studies; data used for all analyses; analytic code; any other materials used in the review. | Page 17 |

*From:* Page MJ, McKenzie JE, Bossuyt PM, Boutron I, Hoffmann TC, Mulrow CD, et al. The PRISMA 2020 statement: an updated guideline for reporting systematic reviews. BMJ 2021;372:n71. doi: 10.1136/bmj.n71

**Table S2.** Search strategy for databases.

**PubMed**

#1 "non-steroidal mineralocorticoid receptor antagonists"[Title/Abstract] OR "nsMRA"[Title/Abstract] OR "mineralocorticoid receptor antagonists"[Title/Abstract] OR "MRA"[Title/Abstract] OR "finerenone"[Title/Abstract] OR "BAY 94-8862"[Title/Abstract] OR "apararenone"[Title/Abstract] OR "MT-3995"[Title/Abstract] OR "esaxerenone"[Title/Abstract] OR "CS-3150"[Title/Abstract] OR "Ocedurenone"[Title/Abstract] OR "KBP-5074"[Title/Abstract] OR "balcinrenone"[Title/Abstract] OR "AZD9977"[Title/Abstract]

#2 "Diabetic Nephropathies"[Mesh]

#3 "diabetic kidney disease"[Title/Abstract] OR "DKD"[Title/Abstract] OR "diabetic nephropathy"[Title/Abstract] OR "DN"[Title/Abstract] OR "chronic kidney disease with type 2 diabetes"[Title/Abstract] OR "chronic kidney disease in type 2 disease"[Title/Abstract] OR "chronic kidney disease associated with type 2 diabetes"[Title/Abstract]

#4 #2 OR #3

#5 #1 AND #4

#6 #5 AND 1900:2026/07/31[dp]

| Search | Actions | Details | Query | Results | Time |
|---|---|---|---|---|---|
| #6 | ••• | ! > | Search: **#5 AND 1900:2026/07/31[dp]** Sort by: **Most Recent** | 615 | 20:32:25 |
| #5 | ••• | ! > | Search: **#1 AND #4** Sort by: **Most Recent** | 628 | 20:32:14 |
| #4 | ••• | ! > | Search: **#2 OR #3** Sort by: **Most Recent** | 59,203 | 20:32:04 |
| #3 | ••• | ! > | Search: **"diabetic kidney disease"[Title/Abstract] OR "DKD"[Title/Abstract] OR "diabetic nephropathy"[Title/Abstract] OR "DN"[Title/Abstract] OR "chronic kidney disease with type 2 diabetes"[Title/Abstract] OR "chronic kidney disease in type 2 disease"[Title/Abstract] OR "chronic kidney disease associated with type 2 diabetes"[Title/Abstract]** Sort by: **Most Recent** | 45,697 | 20:31:52 |
| #2 | ••• | > | Search: **"Diabetic Nephropathies"[Mesh]** Sort by: **Most Recent** | 34,069 | 20:31:39 |
| #1 | ••• | > | Search: **"non-steroidal mineralocorticoid receptor antagonists"[Title/Abstract] OR "nsMRA"[Title/Abstract] OR "mineralocorticoid receptor antagonists"[Title/Abstract] OR "MRA"[Title/Abstract] OR "finerenone"[Title/Abstract] OR "BAY 94-8862"[Title/Abstract] OR "apararenone"[Title/Abstract] OR "MT-3995"[Title/Abstract] OR "esaxerenone"[Title/Abstract] OR "CS-3150"[Title/Abstract] OR "Ocedurenone"[Title/Abstract] OR "KBP-5074"[Title/Abstract] OR "balcinrenone"[Title/Abstract] OR "AZD9977"[Title/Abstract]** Sort by: **Most Recent** | 14,875 | 20:31:25 |

**Embase**

#1 ('non-steroidal mineralocorticoid receptor antagonists': ti, ab, kw OR 'nsmra': ti, ab, kw OR 'mineralocorticoid receptor antagonists': ti, ab, kw OR 'MRA': ti, ab, kw OR 'finerenone': ti, ab, kw OR 'BAY 94-8862': ti, ab, kw OR 'apararenone': ti, ab, kw OR 'MT-3995': ti, ab, kw OR 'esaxerenone': ti, ab, kw OR 'CS-3150': ti, ab, kw OR 'ocedurenone': ti, ab, kw OR 'KBP-5074': ti, ab, kw OR 'balcinrenone': ti, ab, kw OR 'AZD9977': ti, ab, kw)

#2 ('diabetic kidney disease'/exp OR 'diabetic nephropathy'/exp OR 'diabetic kidney disease': ti, ab, kw OR 'DKD': ti, ab, kw OR 'diabetic nephropathy': ti, ab, kw OR 'DN': ti, ab, kw OR 'chronic kidney disease with type 2 diabetes': ti, ab, kw OR 'chronic kidney disease in type 2 disease': ti, ab, kw OR 'chronic kidney disease associated with type 2 diabetes': ti, ab, kw)

#3 #1 AND #2

#4 [01-01-1900]/sd NOT [31-07-2026]/sd

#5 #3 AND #4

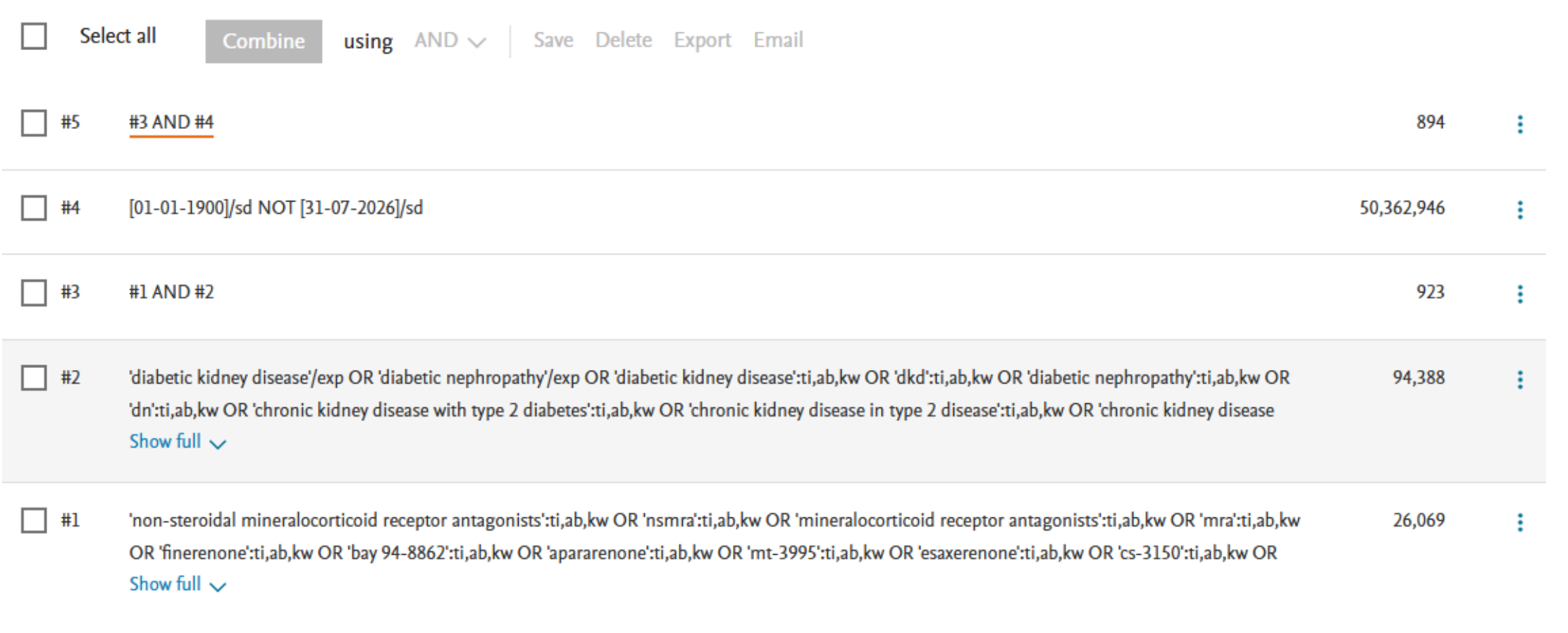


**Web of Science**

#1 TS=("non-steroidal mineralocorticoid receptor antagonists" OR "nsMRA" OR "mineralocorticoid receptor antagonists" OR "MRA" OR "finerenone" OR "BAY 94-8862" OR "apararenone" OR "MT-3995" OR "esaxerenone" OR "CS-3150" OR "Ocedurenone" OR "KBP-5074" OR "balcinreone" OR "AZD9977")

#2 TS=("diabetic kidney disease" OR "DKD" OR "diabetic nephropathy" OR "DN" OR "chronic kidney disease with type 2 diabetes" OR "chronic kidney disease in type 2 disease" OR "chronic kidney disease associated with type 2 diabetes")

#3 #1 AND #2

#4 DOP=(1900-01-01/2026-07-31)

#5 #3 AND #4

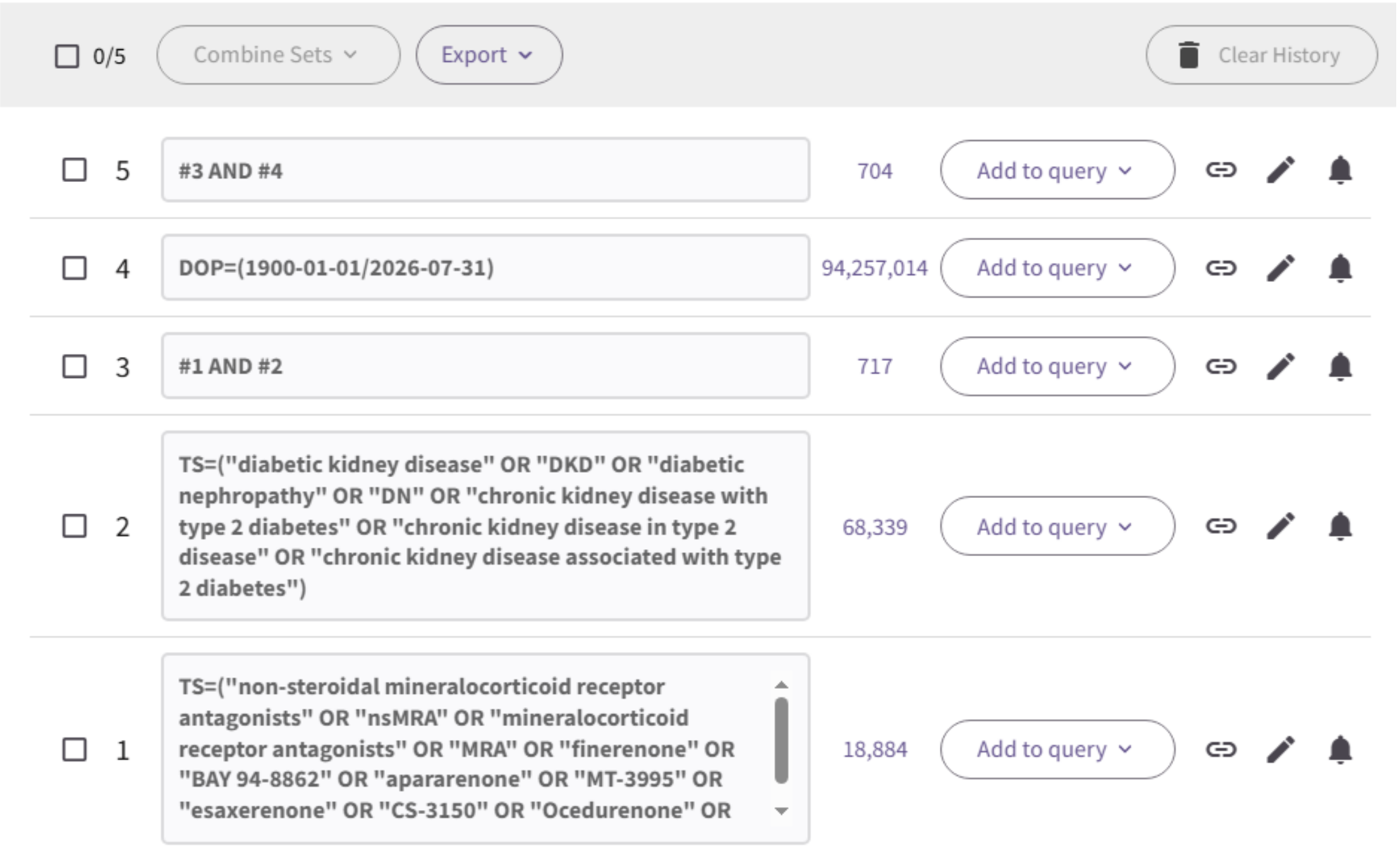


**Cochrane Library**

#1 ("non-steroidal mineralocorticoid receptor antagonists": ti, ab, kw OR "nsMRA": ti, ab, kw OR "mineralocorticoid receptor antagonists": ti, ab, kw OR "MRA": ti, ab, kw OR "finerenone": ti, ab, kw OR "BAY 94-8862": ti, ab, kw OR "apararenone": ti, ab, kw OR "MT-3995": ti, ab, kw OR "esaxerenone": ti, ab, kw OR "CS-3150": ti, ab, kw OR "Ocedurenone": ti, ab, kw OR

"KBP-5074": ti, ab, kw OR "balcinreone": ti, ab, kw OR "AZD9977": ti, ab, kw)

#2 ("diabetic kidney disease": ti, ab, kw OR "DKD": ti, ab, kw OR "diabetic nephropathy": ti, ab, kw OR "DN": ti, ab, kw OR "chronic kidney disease with type 2 diabetes": ti, ab, kw OR "chronic kidney disease in type 2 disease": ti, ab, kw OR "chronic kidney disease associated with type 2 diabetes": ti, ab, kw)

#3 #1 AND #2

#4 Publication Year from 1900 to 2026

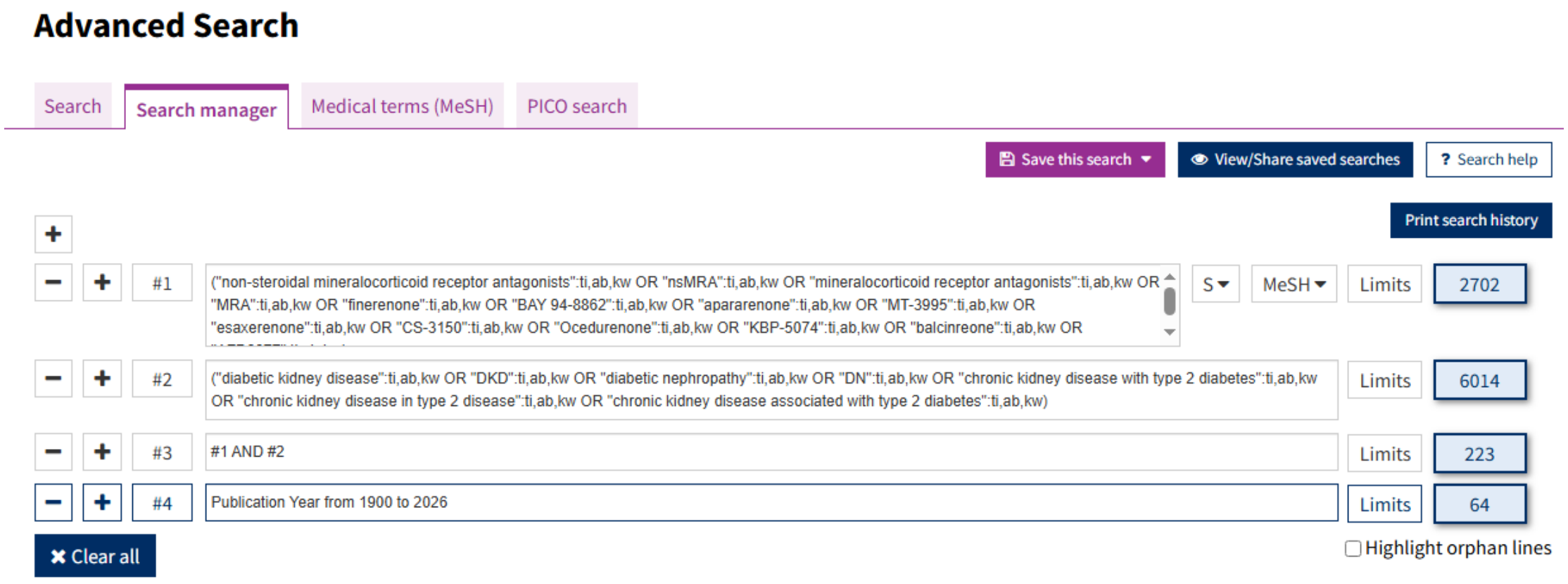

**Table 3** Comparison of the TEAEs between the nsMRAs group and the control group after excluding the short-term trials (follow-up duration less than 24 weeks).

| TEAEs | Studies | nsMRA group (*n*/*N*) | Control group (*n*/*N*) | OR (95% CI) | *P* value（Effect） | Heterogeneity |
|---|---|---|---|---|---|---|
| **Any TEAEs** | 4 | 5787/6787 | 5792/6768 | 0.98 (0.88, 1.07) | P = 0.61 | P = 0.98, $I^2 = 0\%$ |
| Treatment discontinuation due to TEAEs | 5 | 448/7007 | 361/6840 | 1.28 (1.02, 1.61) | P = 0.03 | P = 0.22, $I^2 = 31\%$ |
| drug-related TEAEs | 4 | 1252/6787 | 879/6878 | 1.60 (1.31, 1.97) | P < 0.00001 | P = 0.06, $I^2 = 59\%$ |
| Treatment discontinuation due to drug-related TEAEs* | 1 | 13/226 | 2/209 | 6.93 (1.55, 31.06) | P = 0.01 | NA |
| **Any serious TEAEs** | 4 | 2091/6787 | 2225/6768 | 0.91 (0.84, 0.98) | P = 0.009 | P = 0.89, $I^2 = 0\%$ |
| Treatment discontinuation due to serious TEAEs | 2 | 145/6510 | 154/6489 | 0.94 (0.75, 1.18) | P = 0.58 | P = 0.82, $I^2 = 0\%$ |
| drug-related serious TEAEs | 3 | 84/6561 | 61/6539 | 1.37 (0.99, 1.91) | P = 0.06 | P = 0.86, $I^2 = 0\%$ |

TEAE, treatment-emergent adverse events; n, the number of patients who experienced TEAEs; N, the total number of patients included; NA, not applicable

*Only one study contributed data for this outcome; heterogeneity analysis was not possible, and the result should be interpreted with caution.

**Table S4.** Trial characteristics and subgroup membership for the UACR analysis.

| Trial | Sample size | Baseline UACR<br>mean ± SD/median (range), mg/g | Sample size subgroup membership | Baseline UACR subgroup membership |
|---|---|---|---|---|
| Bakris 2020 | 5674 | Intervention group: 833 (441-1628)<br>Control group: 867 (453-1645) | ≥ 1000 | ≥ 300 mg/g |
| Pitt 2021 | 7352 | Intervention group: 302 (105-749)<br>Control group: 315 (111-731) | ≥ 1000 | ≥ 300 mg/g |
| Bakris 2015 | 821 | Intervention group: 204.9 ± 181.9<br>Control group: 188.4 ± 169.8 | < 1000 | < 300 mg/g |
| Ito 2019 | 358 | Intervention group: 109.3 ± 55.0<br>Control group: 110.0 ± 54.5 | < 1000 | < 300 mg/g |
| Ito 2020 | 449 | Intervention group: 113 (46-286)<br>Control group: 110 (47-278) | < 1000 | < 300 mg/g |
| Katayama 2017 | 96 | Intervention group: 205.94 ± 184.09<br>Control group: 256.80 ± 166.31 | < 1000 | < 300 mg/g |
| Tanaka 2025 | 101 | Intervention group: 221.8 (67.8-533.1)<br>Control group: 190.9 (76.6-470.9) | < 1000 | < 300 mg/g |
| Wada 2021 | 292 | Intervention group: 137.74 ± 82.15<br>Control group: 141.62 ± 88.23 | < 1000 | < 300 mg/g |

UACR, urine albumin-to-creatinine ratio

Footnote: This table presents only the sample size and baseline UACR subgroup variables, which yielded identical subgroup allocations. Other subgroup analyses (age, study duration, drug type, and baseline eGFR) are presented in the corresponding forest plots. Sample size subgroup membership was based on the total sample size of each trial. Baseline UACR subgroup membership was based on the median baseline UACR in the intervention and control arms of each trial; in all included trials, the two arms were concordant with respect to the 300 mg/g threshold.

**Table S5.** Trial characteristics and subgroup membership for the serum potassium analysis.

| Trial | Sample size | Study duration, months | Sample size subgroup membership | Study duration subgroup membership |
|---|---|---|---|---|
| Bakris 2015 | 821 | 3 | ≥ 300 | < 6 months |
| Ito 2019 | 358 | 3 | ≥ 300 | < 6 months |
| Tanaka 2025 | 101 | 6 | < 300 | ≥ 6 months |
| Wada 2021 | 292 | 6 | < 300 | ≥ 6 months |

Footnote: This table presents only the sample size and study duration subgroup variables. Other subgroup analyses (age and drug type) are presented in the corresponding forest plots. Sample size subgroup membership was based on the total sample size of each trial. Study duration subgroup membership was based on the study duration of each trial, which also represented the assessment time point for serum potassium. These two subgroup variables partitioned the same trials into complementary groups.

**Table S6.** Certainty of evidence assessment.

| Certainty assessment | | | | | | | № of patients | | Effect | | Certainty | Importance |
|---|---|---|---|---|---|---|---|---|---|---|---|---|
| № of studies | Study design | Risk of bias | Inconsistency | Indirectness | Imprecision | Other considerations | [Intervention] | [Control] | Relative (95% CI) | Absolute (95% CI) | | |
| **Change in UACR (follow-up: mean 5.13 months)** | | | | | | | | | | | | |
| 14512 | randomised trials | not serious | serious [a] | not serious | serious [b] | none | 7807 | 6705 | - | mean **0.36 lower** (0.44 lower to 0.28 lower) | ⊕⊕◯◯ Low [a, b] | critical |
| **Change in eGFR (follow-up: mean 4 months)** | | | | | | | | | | | | |
| 1218 | randomised trials | not serious | serious [c] | not serious | serious [d] | none | 1005 | 213 | - | mean **3.01 lower** (4.46 lower to 1.56 lower) | ⊕⊕◯◯ Low [c, d] | critical |
| **Change in systolic BP (follow-up: mean 21 months)** | | | | | | | | | | | | |
| 7538 | randomised trials | serious [e] | not serious | not serious | serious [f] | none | 3818 | 3720 | - | mean **4.72 lower** (8.64 lower to 0.8 lower) | ⊕⊕◯◯ Low [e, f] | important |
| **Change in serum potassium (follow-up: mean 4.5 months)** | | | | | | | | | | | | |
| 1468 | randomised trials | serious [g] | not serious | not serious | serious [h] | none | 1192 | 276 | - | mean **0.22 higher** (0.16 higher to 0.29 higher) | ⊕⊕◯◯ Low [g, h] | important |
| **Hyperkalemia (follow-up: mean 22.8 months)** | | | | | | | | | | | | |
| 14734 | randomised trials | not serious | serious [i] | not serious | not serious | none | 958/7800 (12.3%) | 454/6934 (6.5%) | **OR 2.24** (1.99 to 2.52) | **70 more per 1,000** (from 57 more to 85 more) | ⊕⊕⊕◯ Moderate [i] | important |

**CI:** confidence interval; **OR:** odds ratio; **UACR**; urine albumin-to-creatinine ratio; **eGFR**, estimate glomerular filtration rate; **BP**, blood pressure

**Explanations**

a. Downgraded one level for inconsistency. Considerable statistical heterogeneity was present ($I^2 = 86\%$). Although all studies showed a consistent direction of effect, the magnitude varied substantially, reducing confidence in the pooled estimate.

b. Downgraded one level for imprecision. The 95% CI for UACR on the log scale (−0.44 to −0.28) crossed the pre-specified minimal important difference (MID) of −0.36, which corresponds to a 30% reduction based on guideline-recommended thresholds.

c. Downgraded by one level for inconsistency. Clinical heterogeneity arose from methodological differences in eGFR calculation formulas across studies, which could not be fully resolved.

d. Downgraded by one level for imprecision because the 95% CI (-4.46 to -1.56) crossed the calculated MID of 3.7 mL/min/1.73 m2. This was further supported by the sensitivity analysis excluding short-term trials, in which statistical significance was lost although the direction remained consistent, likely reflecting reduced statistical power.

e. Downgraded one level for risk of bias. Of the six studies that reported post-intervention systolic BP means, three studies were excluded due to missing standard deviations (SDs), precluding their inclusion in the meta-analysis. This selective exclusion may have introduced bias.

f. Downgraded one level for imprecision. One study contributed 61.6% of the total meta-analytic weight, meaning the pooled estimate and its 95% CI were effectively driven by a single study. Additionally, the 95% CI (−8.64 to −0.80) crossed the default MID threshold of 7.1 mm Hg and remained relatively wide, further supporting this judgement. The MID was calculated as 0.5 × the median of the baseline SDs from the control arms across all included studies.

g. Downgraded one level for risk of bias. Two large studies were excluded because imputed SD values substantially exceeded the range of the remaining studies and markedly increased heterogeneity ($I^2$ increased from 45% to 78%), suggesting that the imputation introduced artificial between-study heterogeneity. This exclusion may have introduced bias.

h. Downgraded by one level for imprecision. The 95% CI (0.16 to 0.29) crossed the default MID of 0.18 mmol/L, prompting a downgrade. The MID was calculated as 0.5 × the median of the baseline SDs from the control arms across all included studies

i. Downgraded by one level for inconsistency. Diagnostic criteria for hyperkalemia varied across studies (e.g., serum potassium >5.5 mmol/L vs. >6.0 mmol/L), introducing clinical heterogeneity.

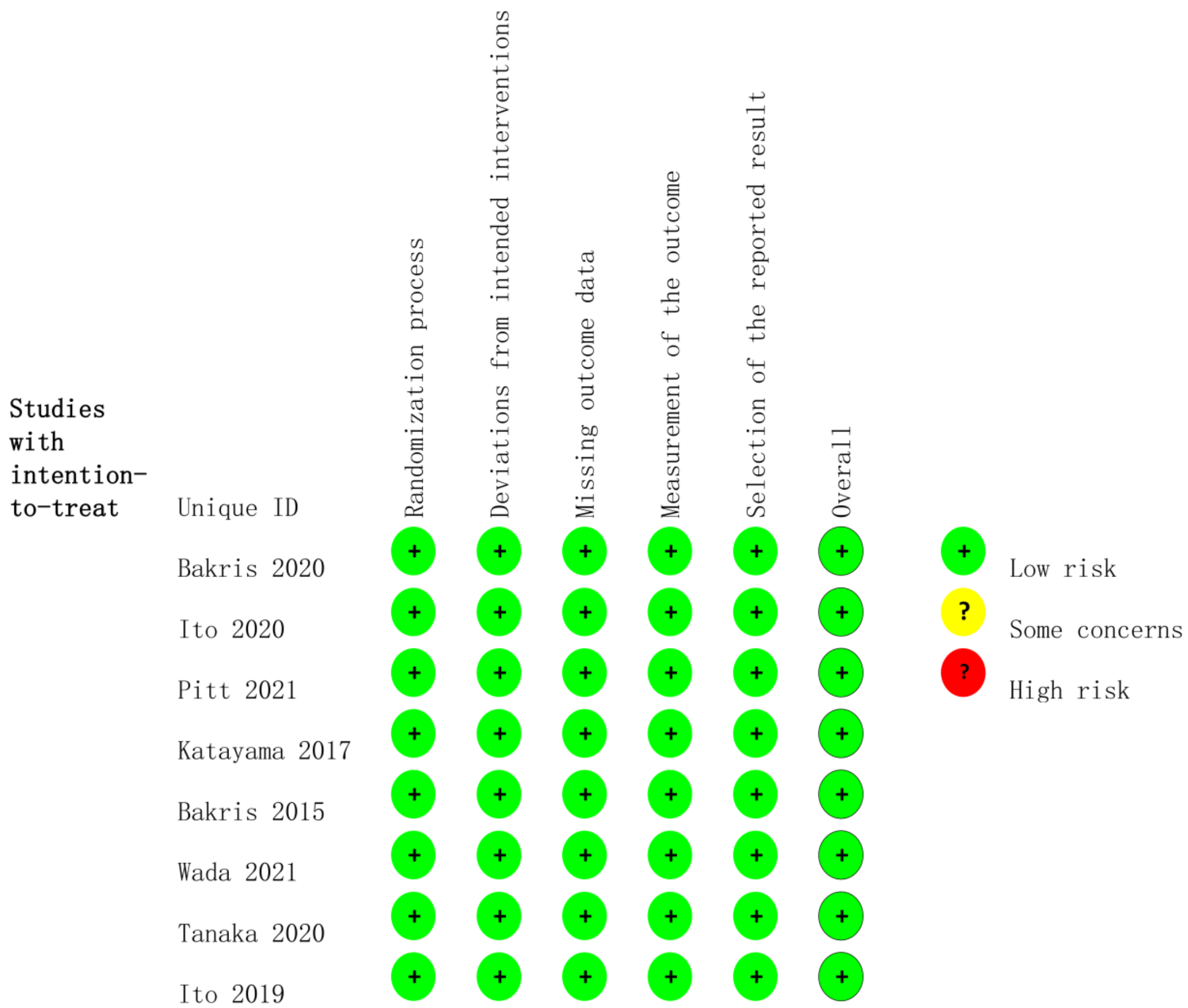


**Figure S1.** Risk of bias in each included study.

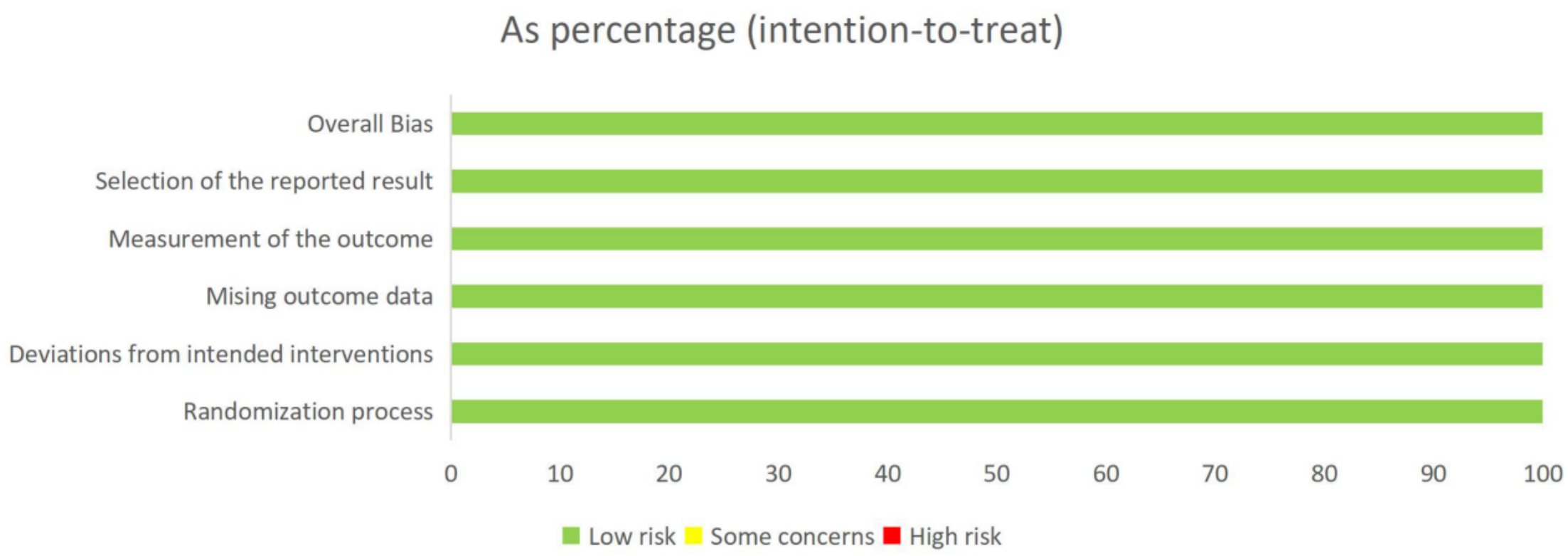


**Figure S2**. Summary of risk of bias for all included studies.

| Study or Subgroup | Experimental Events | Experimental Total | Control Events | Control Total | Weight | Odds Ratio M-H, Fixed, 95% CI |
|---|---|---|---|---|---|---|
| Ito 2019 | 39 | 285 | 2 | 73 | 26.8% | 5.63 [1.33, 23.88] |
| Ito 2020 | 49 | 222 | 9 | 227 | 67.5% | 6.86 [3.28, 14.35] |
| Wada 2021 | 43 | 197 | 0 | 64 | 5.7% | 36.32 [2.20, 598.95] |
| **Total (95% CI)** | | **704** | | **364** | **100.0%** | **8.22 [4.30, 15.69]** |
| Total events | 131 | | 11 | | | |

Heterogeneity: Chi² = 1.57, df = 2 (P = 0.46); I² = 0%
Test for overall effect: Z = 6.38 (P < 0.00001)

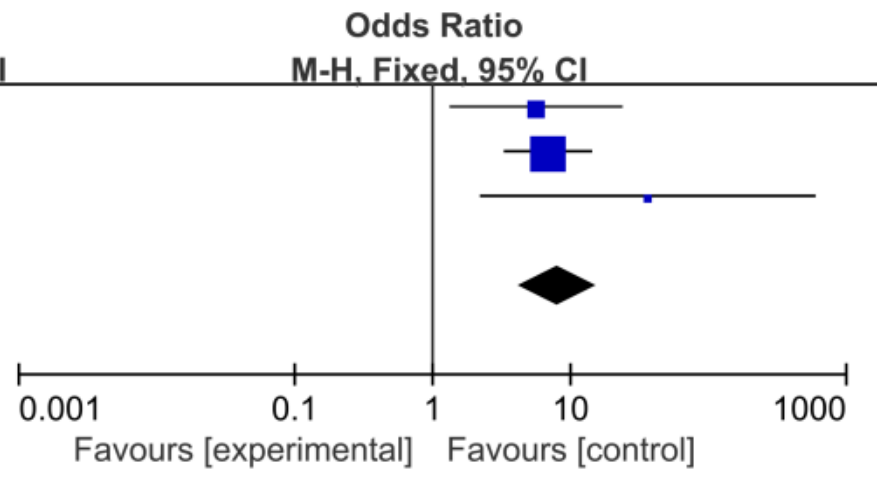


**Figure S3.** Forest plot of the UACR remission rate between the nsMRAs and control groups.

| Study or Subgroup | Experimental Mean | Experimental SD | Experimental Total | Control Mean | Control SD | Control Total | Weight | Mean Difference IV, Random, 95% CI |
|---|---|---|---|---|---|---|---|---|
| Bakris 2015 | 0.142 | 0.39 | 658 | -0.004 | 0.44 | 90 | 12.5% | 0.15 [0.05, 0.24] |
| Bakris 2020 | 0.2 | 0.48 | 2827 | 0.07 | 0.51 | 2831 | 25.8% | 0.13 [0.10, 0.16] |
| Ito 2019 | 0.18 | 0.43 | 286 | -0.1 | 0.3 | 72 | 14.1% | 0.28 [0.19, 0.37] |
| Pitt 2021 | 0.14 | 0.45 | 3683 | 0.03 | 0.47 | 3656 | 26.5% | 0.11 [0.09, 0.13] |
| Tanaka 2025 | 0.2 | 0.36 | 51 | -0.1 | 0.43 | 50 | 6.6% | 0.30 [0.15, 0.45] |
| Wada 2021 | 0.2 | 0.31 | 197 | 0 | 0.29 | 64 | 14.5% | 0.20 [0.12, 0.28] |
| **Total (95% CI)** | | | **7702** | | | **6763** | **100.0%** | **0.17 [0.12, 0.21]** |

Heterogeneity: Tau² = 0.00; Chi² = 22.76, df = 5 (P = 0.0004); I² = 78%
Test for overall effect: Z = 7.30 (P < 0.00001)

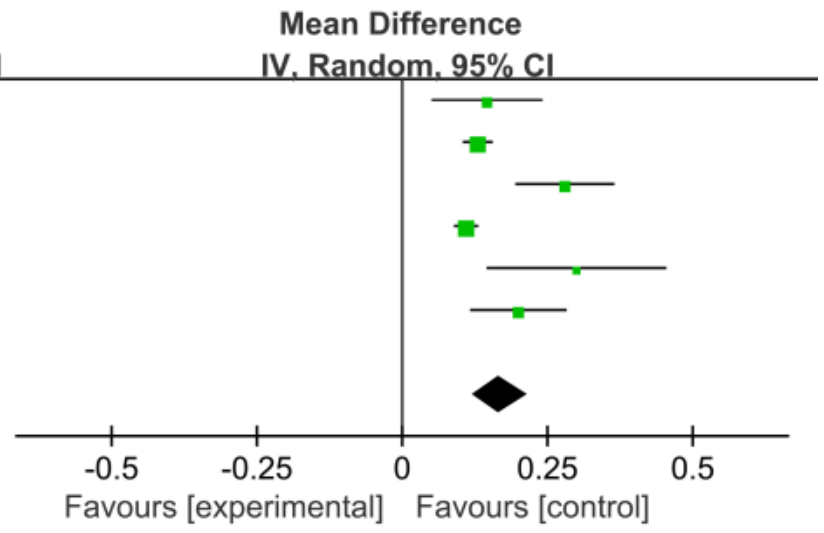


**Figure S4.** Sensitivity analysis: forest plot of change from baseline in serum potassium between the nsMRAs and control groups after including the studies with imputed SDs.

| Study or Subgroup | Experimental Mean | Experimental SD | Experimental Total | Control Mean | Control SD | Control Total | Weight | Mean Difference IV, Random, 95% CI |
|---|---|---|---|---|---|---|---|---|
| Bakris 2015 | 0.142 | 0.39 | 658 | -0.004 | 0.44 | 90 | 13.1% | 0.15 [0.05, 0.24] |
| Bakris 2020 | 0.2 | 0.56 | 2827 | 0.07 | 0.58 | 2831 | 24.6% | 0.13 [0.10, 0.16] |
| Ito 2019 | 0.18 | 0.43 | 286 | -0.1 | 0.3 | 72 | 14.7% | 0.28 [0.19, 0.37] |
| Pitt 2021 | 0.14 | 0.52 | 3683 | 0.03 | 0.55 | 3656 | 25.4% | 0.11 [0.09, 0.13] |
| Tanaka 2025 | 0.2 | 0.36 | 51 | -0.1 | 0.43 | 50 | 7.2% | 0.30 [0.15, 0.45] |
| Wada 2021 | 0.2 | 0.31 | 197 | 0 | 0.29 | 64 | 15.0% | 0.20 [0.12, 0.28] |
| **Total (95% CI)** | | | **7702** | | | **6763** | **100.0%** | **0.17 [0.12, 0.22]** |

Heterogeneity: Tau² = 0.00; Chi² = 21.84, df = 5 (P = 0.0006); I² = 77%
Test for overall effect: Z = 6.97 (P < 0.00001)

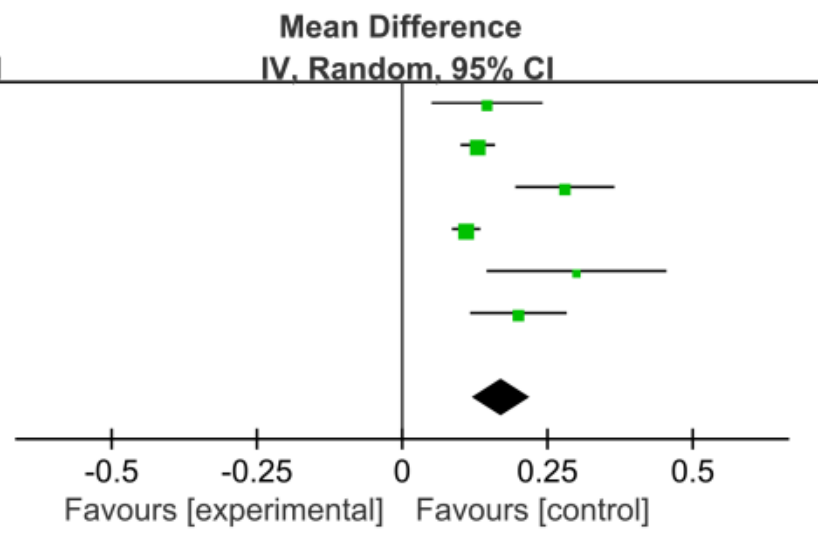


**Figure S5.** Sensitivity analysis: forest plot of change from baseline in serum potassium between the nsMRAs and control groups, using a lower correlation ($r_{mean}$ - 0.2) for imputation of missing SDs.

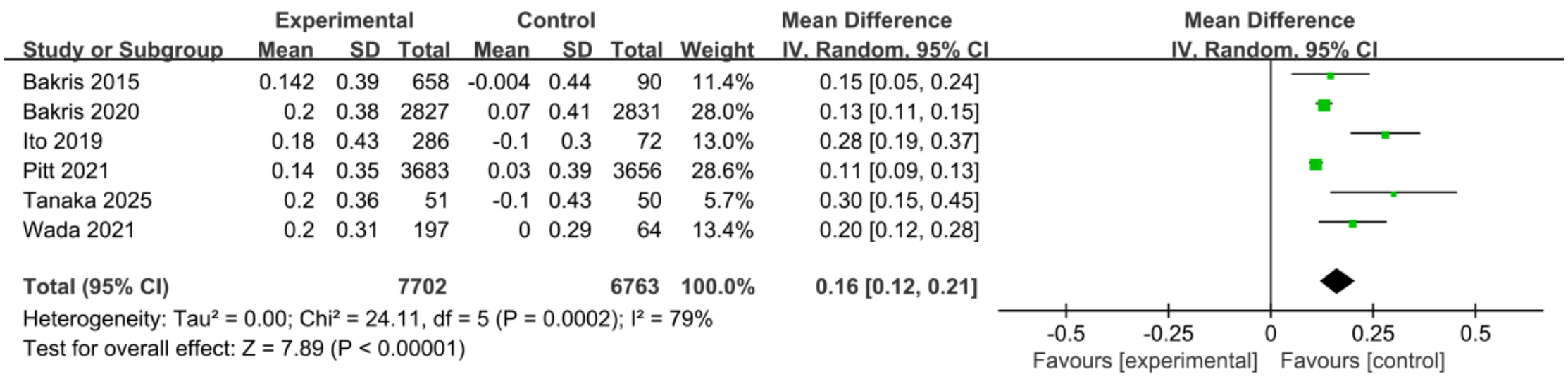


**Figure S6.** Sensitivity analysis: forest plot of change from baseline in serum potassium between the nsMRAs and control groups, using a higher correlation ($r_{mean}$ + 0.2) for imputation of missing SDs.

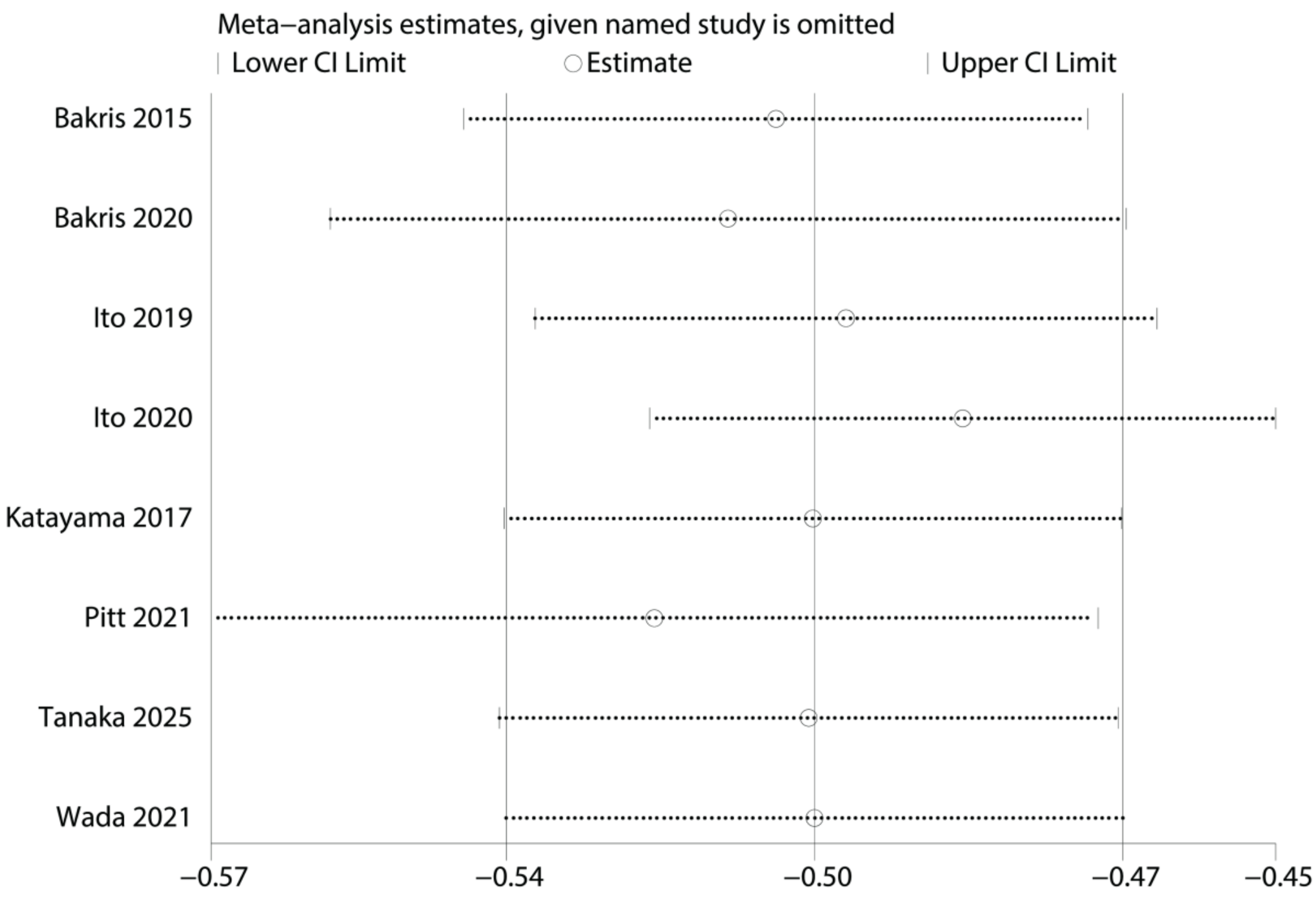


**Figure S7.** Sensitivity analysis using the leave-one-out method for the log-transformed LS mean ratio for UACR between the nsMRAs and control groups.

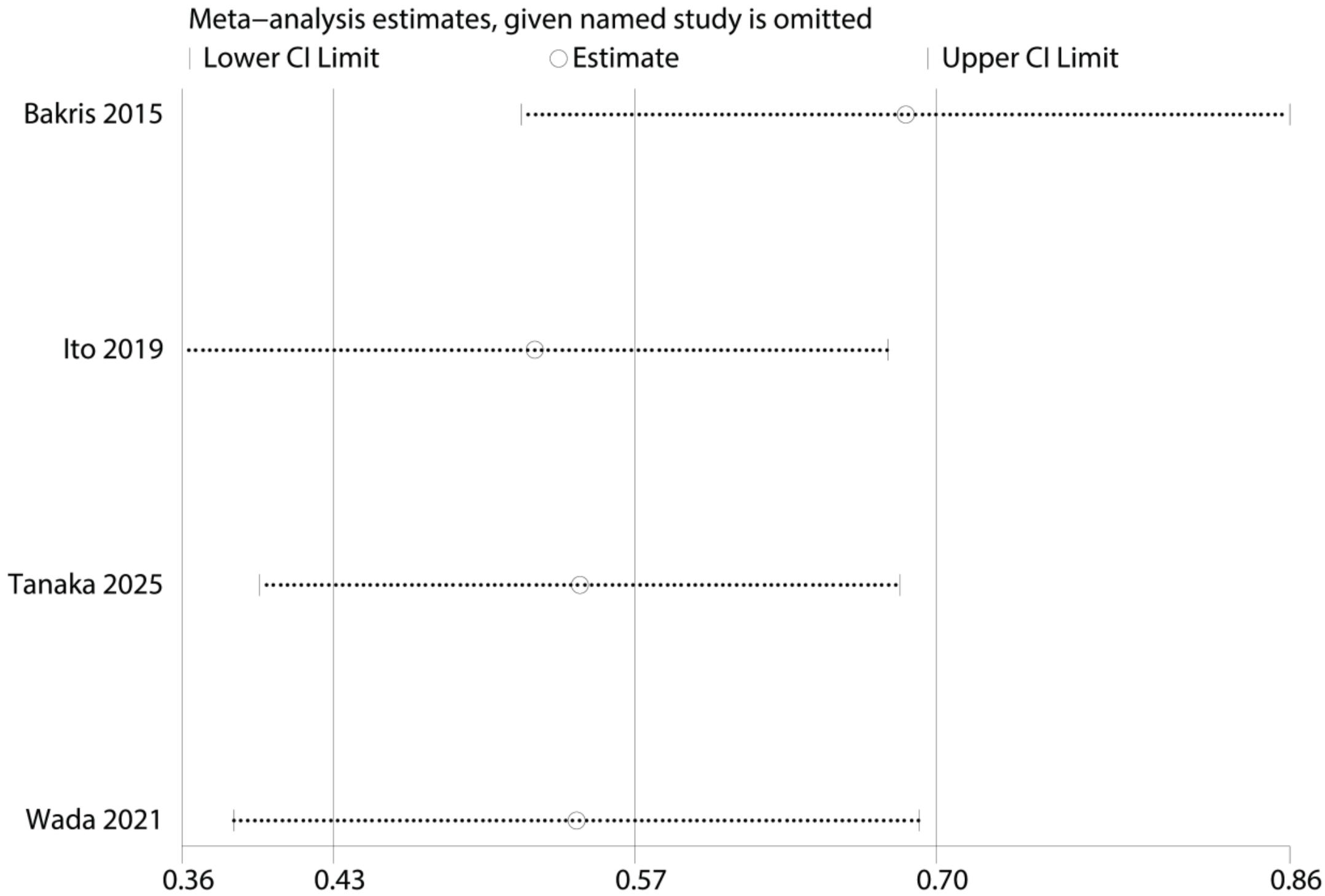


**Figure S8.** Sensitivity analysis using the leave-one-out method for serum potassium between the nsMRAs and control groups.

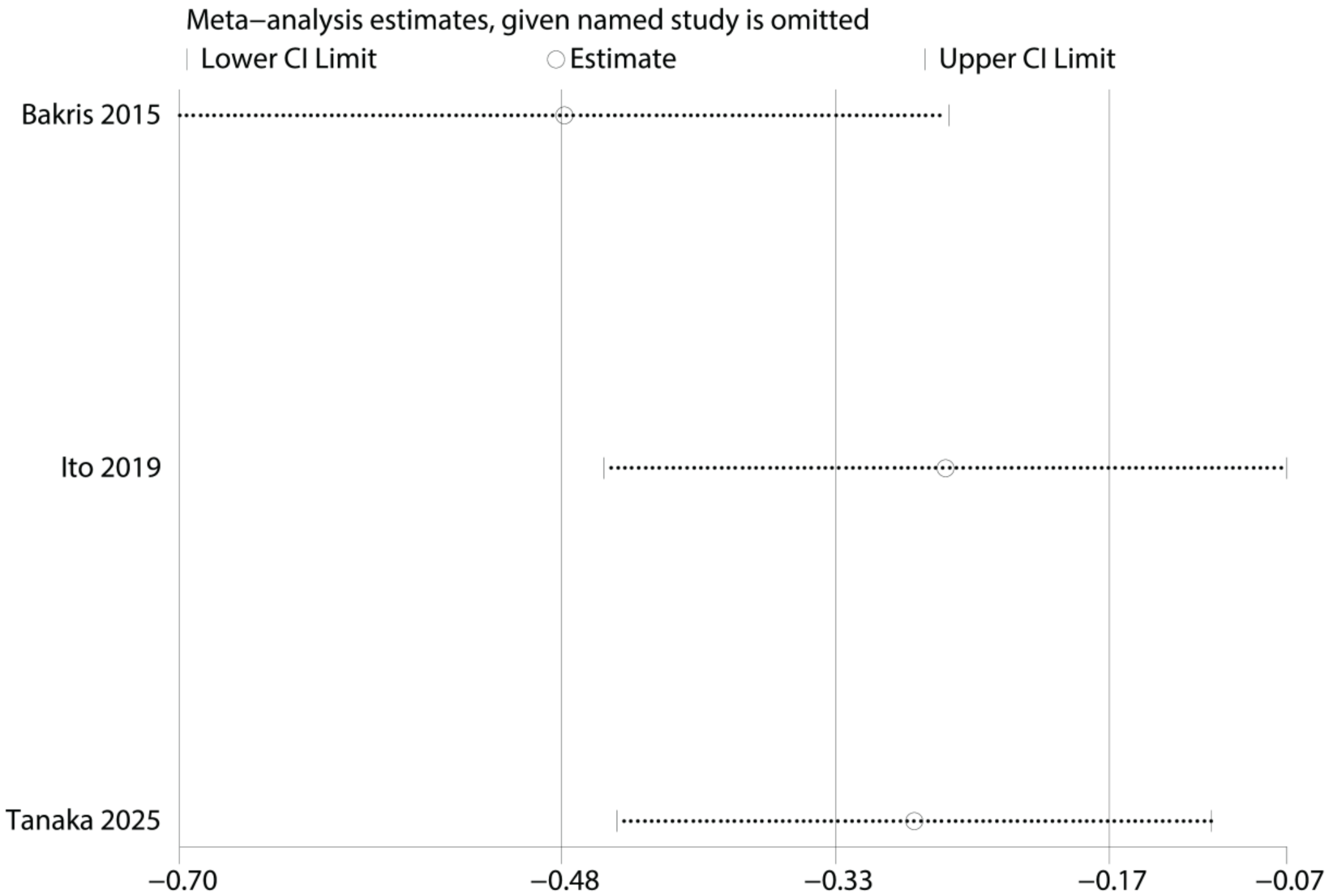


**Figure S9.** Sensitivity analysis using the leave-one-out method for decline in eGFR between the nsMRAs and control groups.

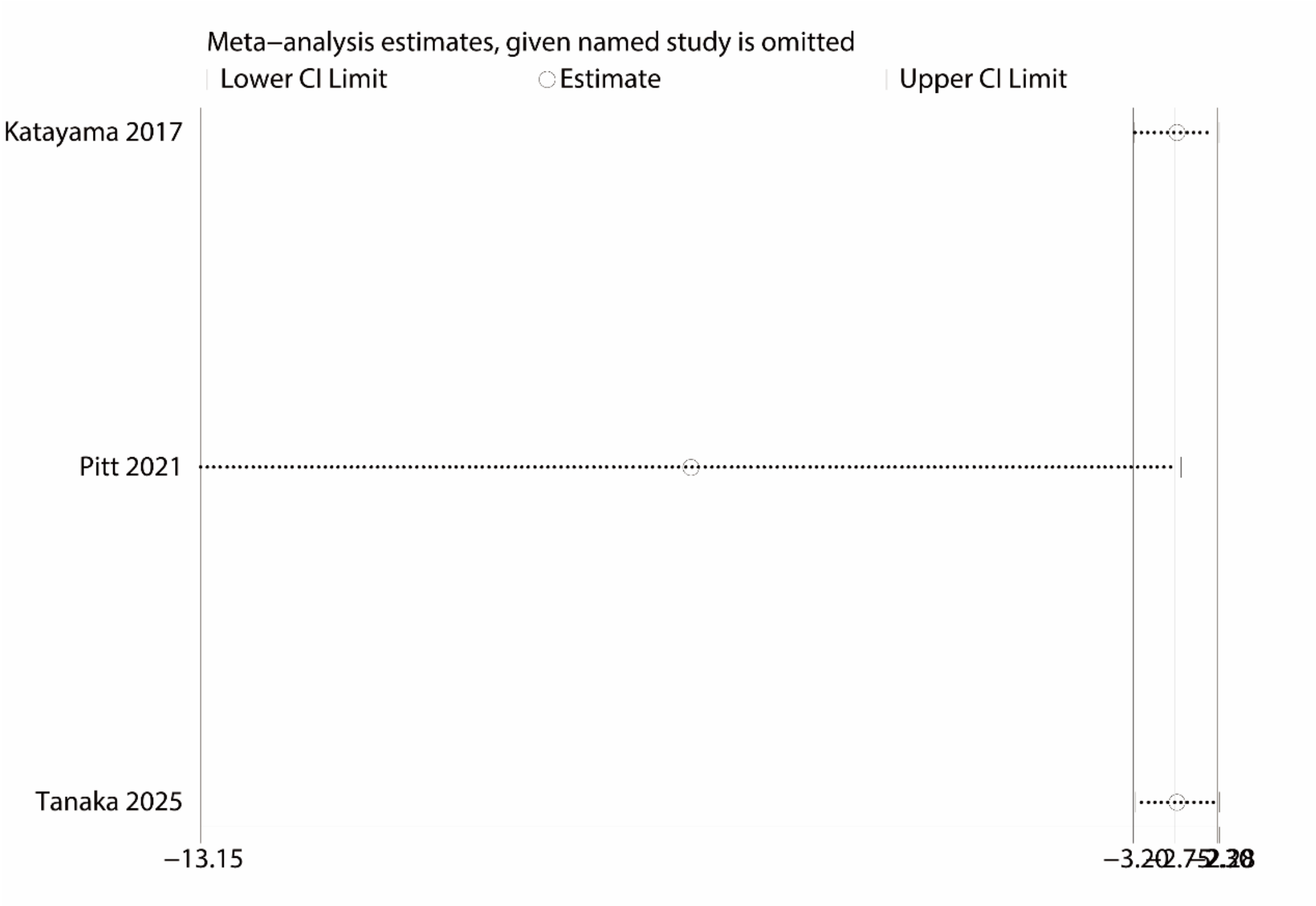


**Figure S10.** Sensitivity analysis using the leave-one-out method for change from baseline in systolic BP between the nsMRAs and control groups.

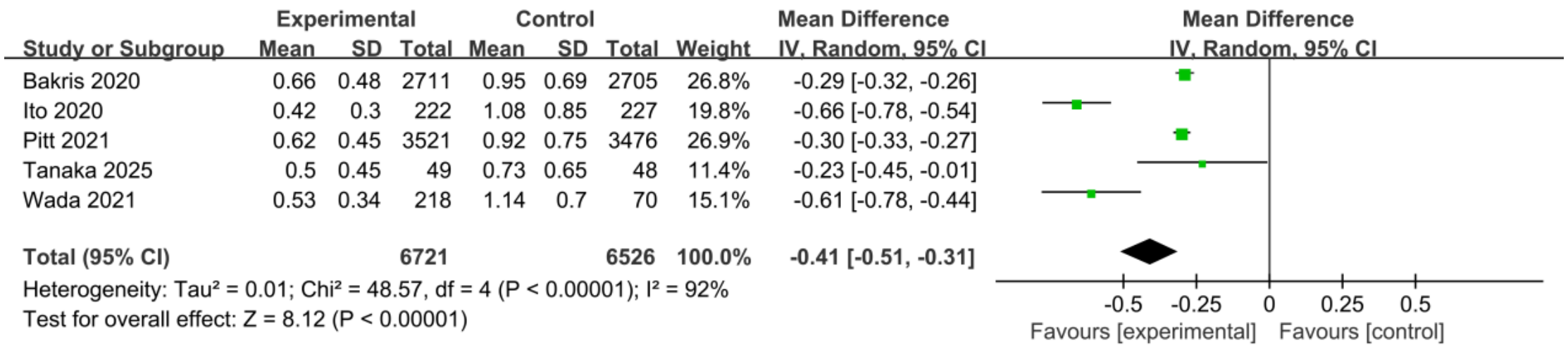

| | Experimental | | | Control | | | | Mean Difference |
|---|---|---|---|---|---|---|---|---|
| Study or Subgroup | Mean | SD | Total | Mean | SD | Total | Weight | IV, Random, 95% CI |
| Bakris 2020 | 0.66 | 0.48 | 2711 | 0.95 | 0.69 | 2705 | 26.8% | -0.29 [-0.32, -0.26] |
| Ito 2020 | 0.42 | 0.3 | 222 | 1.08 | 0.85 | 227 | 19.8% | -0.66 [-0.78, -0.54] |
| Pitt 2021 | 0.62 | 0.45 | 3521 | 0.92 | 0.75 | 3476 | 26.9% | -0.30 [-0.33, -0.27] |
| Tanaka 2025 | 0.5 | 0.45 | 49 | 0.73 | 0.65 | 48 | 11.4% | -0.23 [-0.45, -0.01] |
| Wada 2021 | 0.53 | 0.34 | 218 | 1.14 | 0.7 | 70 | 15.1% | -0.61 [-0.78, -0.44] |
| **Total (95% CI)** | | | **6721** | | | **6526** | **100.0%** | **-0.41 [-0.51, -0.31]** |

Heterogeneity: $Tau^2 = 0.01$; $Chi^2 = 48.57$, df = 4 ($P < 0.00001$); $I^2 = 92\%$
Test for overall effect: Z = 8.12 ($P < 0.00001$)



**Figure S11**. Sensitivity analysis: forest plot of change from baseline in UACR between the nsMRAs and control groups after excluding the short-term trials.

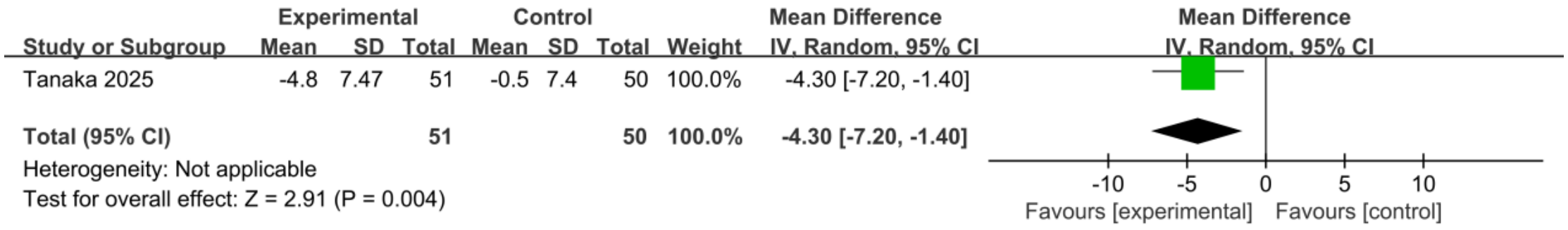


**Figure S12**. Sensitivity analysis: forest plot of change from baseline in eGFR between the nsMRAs and control groups after excluding the short-term trials.

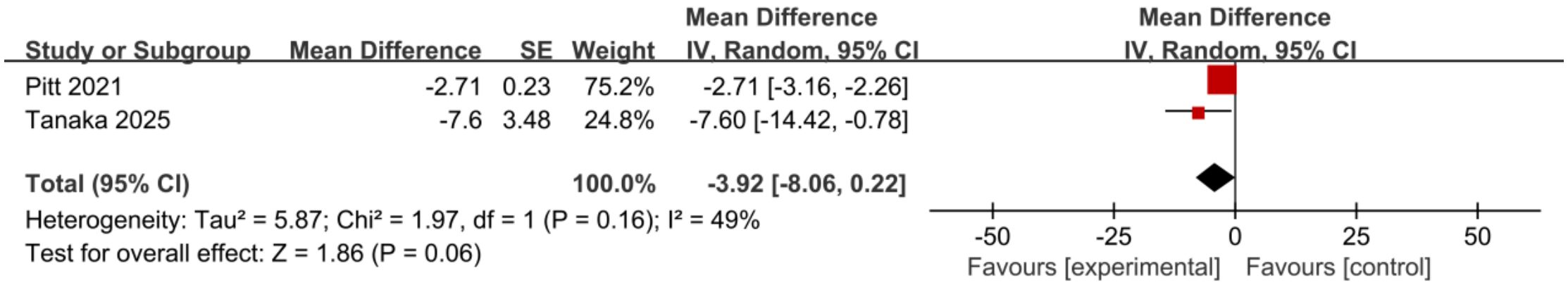


**Figure S13**. Sensitivity analysis: forest plot of change from baseline in systolic BP between the nsMRAs and control groups after excluding the short-term trials.

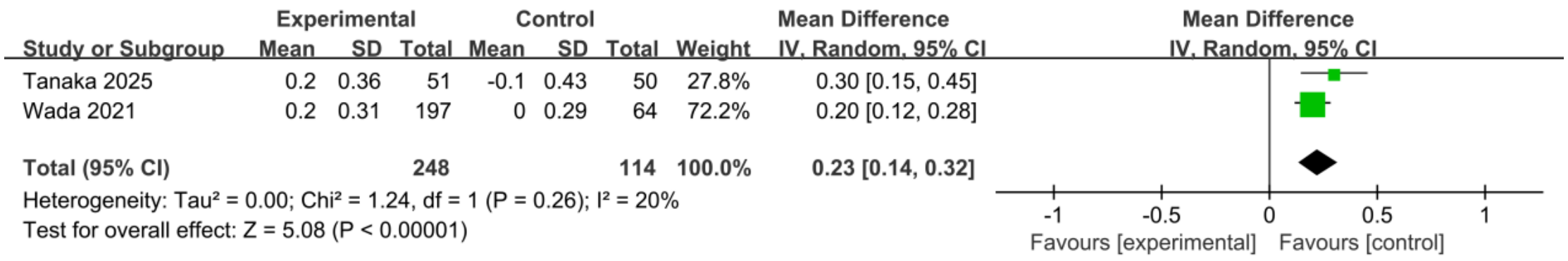


**Figure S14**. Sensitivity analysis: forest plot of change from baseline in serum potassium between the nsMRAs and control groups after excluding the short-term trials.

| Study or Subgroup | Experimental Mean | Experimental SD | Experimental Total | Control Mean | Control SD | Control Total | Weight | Mean Difference IV, Random, 95% CI |
|---|---|---|---|---|---|---|---|---|
| **57.1.1 n ⩾ 1000** | | | | | | | | |
| Bakris 2020 | 0.66 | 0.48 | 2711 | 0.95 | 0.69 | 2705 | 19.6% | -0.29 [-0.32, -0.26] |
| Pitt 2021 | 0.62 | 0.45 | 3521 | 0.92 | 0.75 | 3476 | 19.7% | -0.30 [-0.33, -0.27] |
| **Subtotal (95% CI)** | | | **6232** | | | **6181** | **39.3%** | **-0.30 [-0.32, -0.27]** |
| Heterogeneity: Tau² = 0.00; Chi² = 0.21, df = 1 (P = 0.65); I² = 0% | | | | | | | | |
| Test for overall effect: Z = 27.06 (P < 0.00001) | | | | | | | | |
| **57.1.2 n < 1000** | | | | | | | | |
| Bakris 2015 | 0.75 | 0.55 | 718 | 0.94 | 0.68 | 94 | 12.3% | -0.19 [-0.33, -0.05] |
| Ito 2019 | 0.57 | 0.41 | 285 | 0.9 | 0.65 | 73 | 11.4% | -0.33 [-0.49, -0.17] |
| Ito 2020 | 0.42 | 0.3 | 222 | 1.08 | 0.85 | 227 | 14.1% | -0.66 [-0.78, -0.54] |
| Katayama 2017 | 0.85 | 0.46 | 83 | 1.06 | 0.57 | 12 | 4.4% | -0.21 [-0.55, 0.13] |
| Tanaka 2025 | 0.5 | 0.45 | 49 | 0.73 | 0.65 | 48 | 7.9% | -0.23 [-0.45, -0.01] |
| Wada 2021 | 0.53 | 0.34 | 218 | 1.14 | 0.7 | 70 | 10.6% | -0.61 [-0.78, -0.44] |
| **Subtotal (95% CI)** | | | **1575** | | | **524** | **60.7%** | **-0.38 [-0.57, -0.20]** |
| Heterogeneity: Tau² = 0.05; Chi² = 36.10, df = 5 (P < 0.00001); I² = 86% | | | | | | | | |
| Test for overall effect: Z = 4.00 (P < 0.0001) | | | | | | | | |
| **Total (95% CI)** | | | **7807** | | | **6705** | **100.0%** | **-0.36 [-0.44, -0.28]** |
| Heterogeneity: Tau² = 0.01; Chi² = 51.65, df = 7 (P < 0.00001); I² = 86% | | | | | | | | |
| Test for overall effect: Z = 8.86 (P < 0.00001) | | | | | | | | |
| Test for subgroup differences: Chi² = 0.85, df = 1 (P = 0.36), I² = 0% | | | | | | | | |

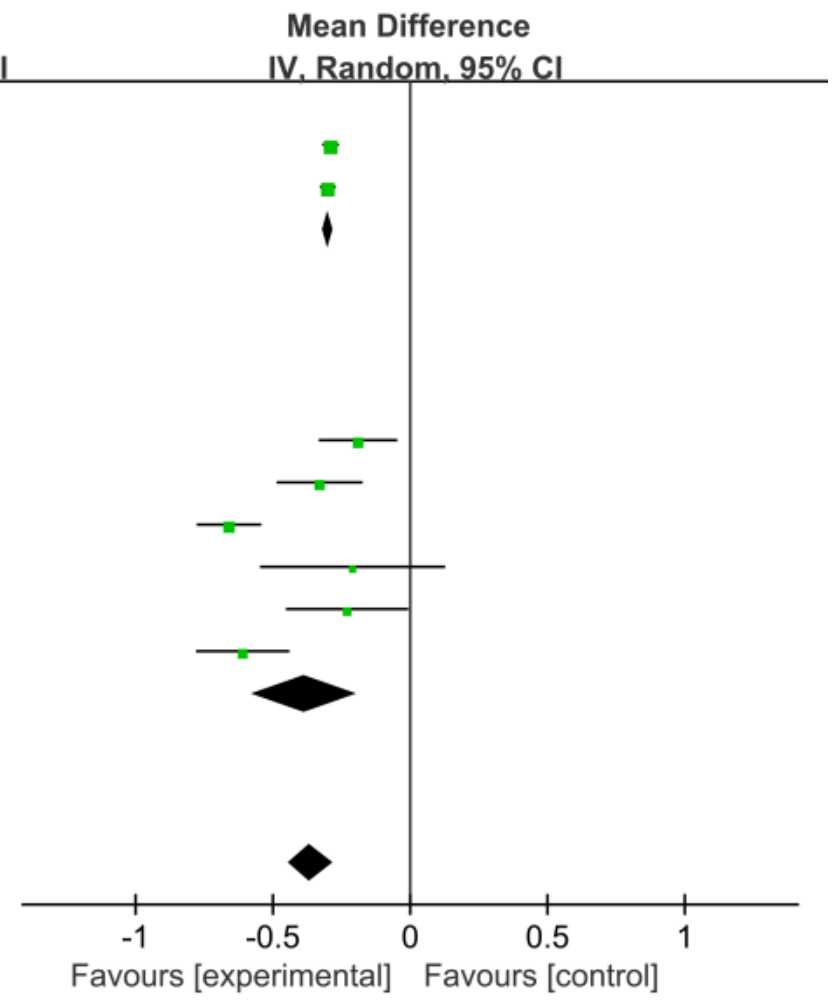


**Figure S15.** Subgroup analysis of the log-transformed LS mean ratio for UACR between the nsMRAs and control groups, stratified by sample size.

| Study or Subgroup | Experimental Mean | Experimental SD | Experimental Total | Control Mean | Control SD | Control Total | Weight | Mean Difference IV, Random, 95% CI |
|---|---|---|---|---|---|---|---|---|
| **62.1.1 ⩾ 65 years** | | | | | | | | |
| Bakris 2020 | 0.66 | 0.48 | 2711 | 0.95 | 0.69 | 2705 | 20.4% | -0.29 [-0.32, -0.26] |
| Ito 2019 | 0.57 | 0.41 | 285 | 0.9 | 0.65 | 73 | 12.0% | -0.33 [-0.49, -0.17] |
| Ito 2020 | 0.42 | 0.3 | 222 | 1.08 | 0.85 | 227 | 14.8% | -0.66 [-0.78, -0.54] |
| Tanaka 2025 | 0.5 | 0.45 | 49 | 0.73 | 0.65 | 48 | 8.3% | -0.23 [-0.45, -0.01] |
| **Subtotal (95% CI)** | | | **3267** | | | **3053** | **55.5%** | **-0.38 [-0.58, -0.19]** |
| Heterogeneity: Tau² = 0.04; Chi² = 36.16, df = 3 (P < 0.00001); I² = 92% | | | | | | | | |
| Test for overall effect: Z = 3.81 (P = 0.0001) | | | | | | | | |
| **62.1.2 < 65 years** | | | | | | | | |
| Bakris 2015 | 0.75 | 0.55 | 718 | 0.94 | 0.68 | 94 | 12.9% | -0.19 [-0.33, -0.05] |
| Pitt 2021 | 0.62 | 0.45 | 3521 | 0.92 | 0.75 | 3476 | 20.5% | -0.30 [-0.33, -0.27] |
| Wada 2021 | 0.53 | 0.34 | 218 | 1.14 | 0.7 | 70 | 11.1% | -0.61 [-0.78, -0.44] |
| **Subtotal (95% CI)** | | | **4457** | | | **3640** | **44.5%** | **-0.36 [-0.54, -0.18]** |
| Heterogeneity: Tau² = 0.02; Chi² = 14.94, df = 2 (P = 0.0006); I² = 87% | | | | | | | | |
| Test for overall effect: Z = 3.88 (P = 0.0001) | | | | | | | | |
| **Total (95% CI)** | | | **7724** | | | **6693** | **100.0%** | **-0.37 [-0.45, -0.29]** |
| Heterogeneity: Tau² = 0.01; Chi² = 51.32, df = 6 (P < 0.00001); I² = 88% | | | | | | | | |
| Test for overall effect: Z = 8.76 (P < 0.00001) | | | | | | | | |
| Test for subgroup differences: Chi² = 0.04, df = 1 (P = 0.84), I² = 0% | | | | | | | | |

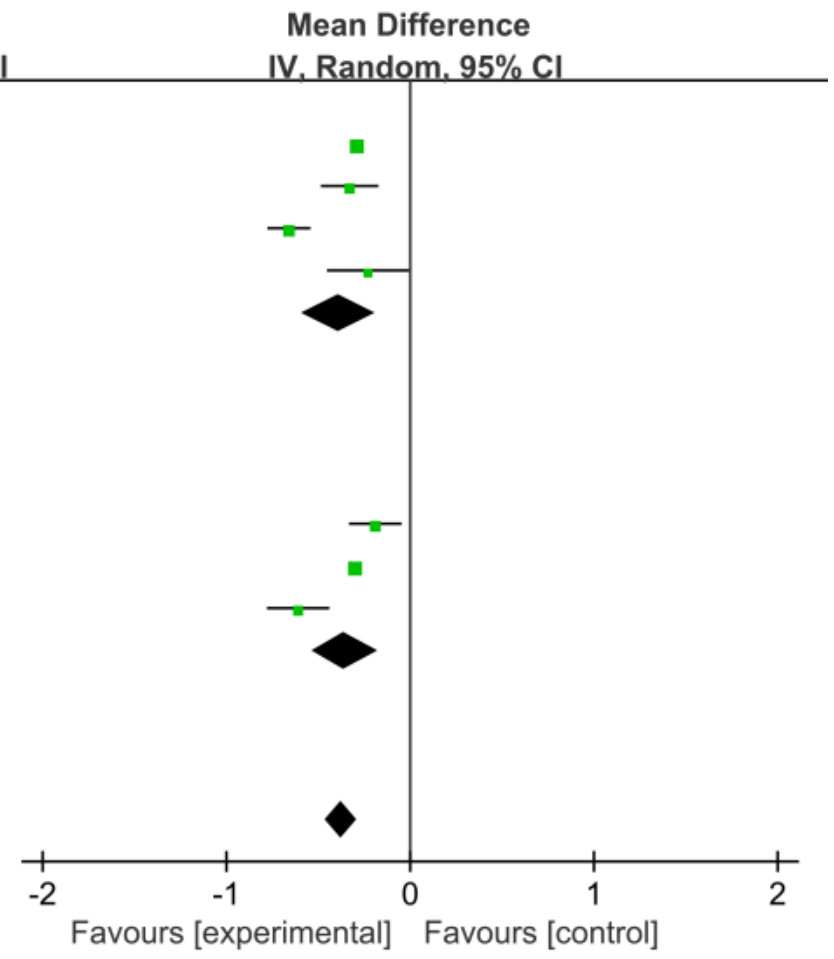


**Figure S16.** Subgroup analysis of the log-transformed LS mean ratio for UACR between the nsMRAs and control groups, stratified by age.

| Study or Subgroup | Experimental Mean | Experimental SD | Experimental Total | Control Mean | Control SD | Control Total | Weight | Mean Difference IV, Random, 95% CI |
|---|---|---|---|---|---|---|---|---|
| **58.1.1 ⩾ 6 months** | | | | | | | | |
| Ito 2020 | 0.42 | 0.3 | 222 | 1.08 | 0.85 | 227 | 14.1% | -0.66 [-0.78, -0.54] |
| Tanaka 2025 | 0.5 | 0.45 | 49 | 0.73 | 0.65 | 48 | 7.9% | -0.23 [-0.45, -0.01] |
| Wada 2021 | 0.53 | 0.34 | 218 | 1.14 | 0.7 | 70 | 10.6% | -0.61 [-0.78, -0.44] |
| **Subtotal (95% CI)** | | | **489** | | | **345** | **32.6%** | **-0.52 [-0.75, -0.29]** |
| Heterogeneity: Tau² = 0.03; Chi² = 11.37, df = 2 (P = 0.003); I² = 82% | | | | | | | | |
| Test for overall effect: Z = 4.42 (P < 0.00001) | | | | | | | | |
| **58.1.2 < 6 months** | | | | | | | | |
| Bakris 2015 | 0.75 | 0.55 | 718 | 0.94 | 0.68 | 94 | 12.3% | -0.19 [-0.33, -0.05] |
| Bakris 2020 | 0.66 | 0.48 | 2711 | 0.95 | 0.69 | 2705 | 19.6% | -0.29 [-0.32, -0.26] |
| Ito 2019 | 0.57 | 0.41 | 285 | 0.9 | 0.65 | 73 | 11.4% | -0.33 [-0.49, -0.17] |
| Katayama 2017 | 0.85 | 0.46 | 83 | 1.06 | 0.57 | 12 | 4.4% | -0.21 [-0.55, 0.13] |
| Pitt 2021 | 0.62 | 0.45 | 3521 | 0.92 | 0.75 | 3476 | 19.7% | -0.30 [-0.33, -0.27] |
| **Subtotal (95% CI)** | | | **7318** | | | **6360** | **67.4%** | **-0.29 [-0.31, -0.27]** |
| Heterogeneity: Tau² = 0.00; Chi² = 2.69, df = 4 (P = 0.61); I² = 0% | | | | | | | | |
| Test for overall effect: Z = 27.48 (P < 0.00001) | | | | | | | | |
| **Total (95% CI)** | | | **7807** | | | **6705** | **100.0%** | **-0.36 [-0.44, -0.28]** |
| Heterogeneity: Tau² = 0.01; Chi² = 51.65, df = 7 (P < 0.00001); I² = 86% | | | | | | | | |
| Test for overall effect: Z = 8.86 (P < 0.00001) | | | | | | | | |
| Test for subgroup differences: Chi² = 3.61, df = 1 (P = 0.06), I² = 72.3% | | | | | | | | |

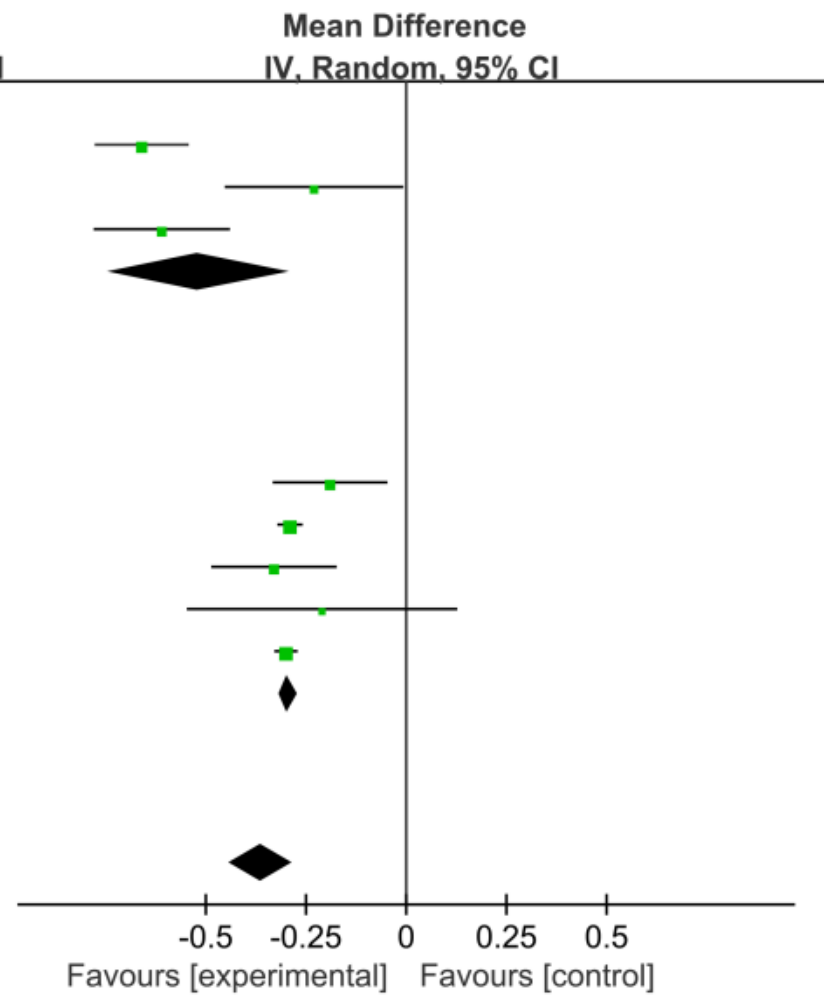


**Figure S17.** Subgroup analysis of the log-transformed LS mean ratio for UACR between the nsMRAs and control groups, stratified by study duration.

| Study or Subgroup | Experimental Mean | Experimental SD | Experimental Total | Control Mean | Control SD | Control Total | Weight | Mean Difference IV, Random, 95% CI |
|---|---|---|---|---|---|---|---|---|
| **60.1.1 ⩾ 300 mg/g** | | | | | | | | |
| Bakris 2020 | 0.66 | 0.48 | 2711 | 0.95 | 0.69 | 2705 | 19.6% | -0.29 [-0.32, -0.26] |
| Pitt 2021 | 0.62 | 0.45 | 3521 | 0.92 | 0.75 | 3476 | 19.7% | -0.30 [-0.33, -0.27] |
| **Subtotal (95% CI)** | | | **6232** | | | **6181** | **39.3%** | **-0.30 [-0.32, -0.27]** |
| Heterogeneity: Tau² = 0.00; Chi² = 0.21, df = 1 (P = 0.65); I² = 0% | | | | | | | | |
| Test for overall effect: Z = 27.06 (P < 0.00001) | | | | | | | | |
| **60.1.2 < 300 mg/g** | | | | | | | | |
| Bakris 2015 | 0.75 | 0.55 | 718 | 0.94 | 0.68 | 94 | 12.3% | -0.19 [-0.33, -0.05] |
| Ito 2019 | 0.57 | 0.41 | 285 | 0.9 | 0.65 | 73 | 11.4% | -0.33 [-0.49, -0.17] |
| Ito 2020 | 0.42 | 0.3 | 222 | 1.08 | 0.85 | 227 | 14.1% | -0.66 [-0.78, -0.54] |
| Katayama 2017 | 0.85 | 0.46 | 83 | 1.06 | 0.57 | 12 | 4.4% | -0.21 [-0.55, 0.13] |
| Tanaka 2025 | 0.5 | 0.45 | 49 | 0.73 | 0.65 | 48 | 7.9% | -0.23 [-0.45, -0.01] |
| Wada 2021 | 0.53 | 0.34 | 218 | 1.14 | 0.7 | 70 | 10.6% | -0.61 [-0.78, -0.44] |
| **Subtotal (95% CI)** | | | **1575** | | | **524** | **60.7%** | **-0.38 [-0.57, -0.20]** |
| Heterogeneity: Tau² = 0.05; Chi² = 36.10, df = 5 (P < 0.00001); I² = 86% | | | | | | | | |
| Test for overall effect: Z = 4.00 (P < 0.0001) | | | | | | | | |
| **Total (95% CI)** | | | **7807** | | | **6705** | **100.0%** | **-0.36 [-0.44, -0.28]** |
| Heterogeneity: Tau² = 0.01; Chi² = 51.65, df = 7 (P < 0.00001); I² = 86% | | | | | | | | |
| Test for overall effect: Z = 8.86 (P < 0.00001) | | | | | | | | |
| Test for subgroup differences: Chi² = 0.85, df = 1 (P = 0.36), I² = 0% | | | | | | | | |

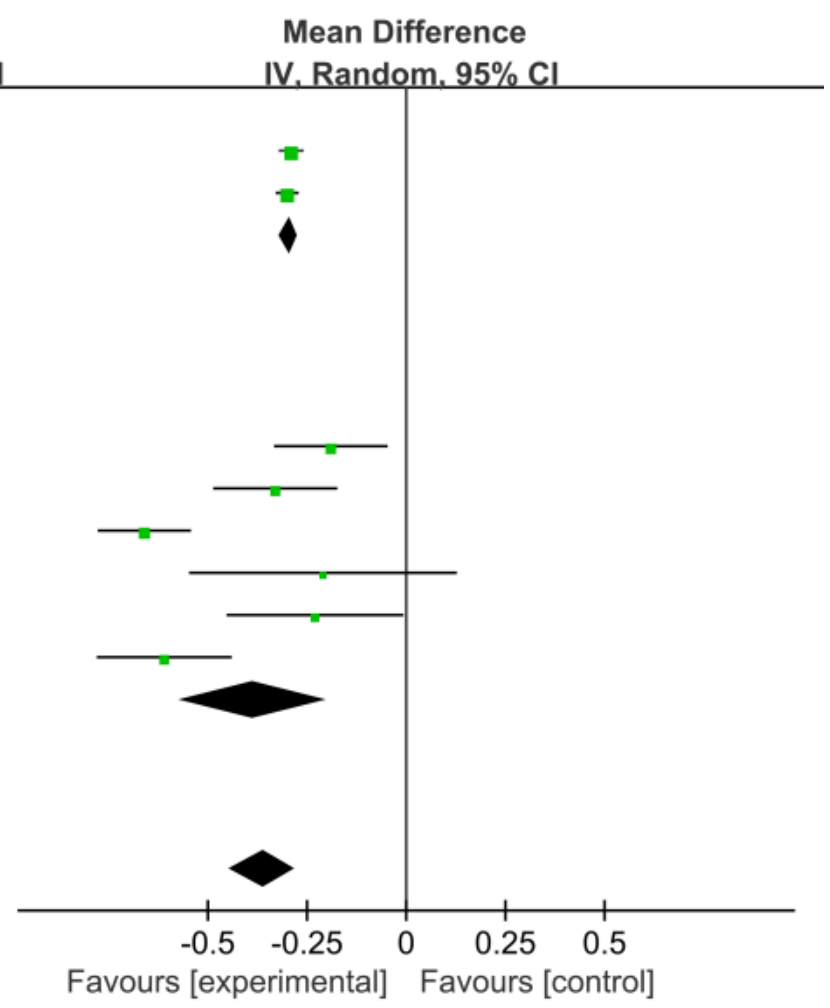


**Figure S18.** Subgroup analysis of the log-transformed LS mean ratio for UACR between the nsMRAs and control groups, stratified by baseline UACR levels of participants.

| Study or Subgroup | Experimental Mean | Experimental SD | Experimental Total | Control Mean | Control SD | Control Total | Weight | Mean Difference IV, Random, 95% CI |
|---|---|---|---|---|---|---|---|---|
| **59.1.1 ⩾ 60 ml/min/1.73 m2** | | | | | | | | |
| Bakris 2015 | 0.75 | 0.55 | 718 | 0.94 | 0.68 | 94 | 12.3% | -0.19 [-0.33, -0.05] |
| Ito 2019 | 0.57 | 0.41 | 285 | 0.9 | 0.65 | 73 | 11.4% | -0.33 [-0.49, -0.17] |
| Ito 2020 | 0.42 | 0.3 | 222 | 1.08 | 0.85 | 227 | 14.1% | -0.66 [-0.78, -0.54] |
| Katayama 2017 | 0.85 | 0.46 | 83 | 1.06 | 0.57 | 12 | 4.4% | -0.21 [-0.55, 0.13] |
| Pitt 2021 | 0.62 | 0.45 | 3521 | 0.92 | 0.75 | 3476 | 19.7% | -0.30 [-0.33, -0.27] |
| Wada 2021 | 0.53 | 0.34 | 218 | 1.14 | 0.7 | 70 | 10.6% | -0.61 [-0.78, -0.44] |
| **Subtotal (95% CI)** | | | **5047** | | | **3952** | **72.5%** | **-0.39 [-0.55, -0.24]** |
| Heterogeneity: Tau² = 0.03; Chi² = 48.75, df = 5 (P < 0.00001); I² = 90% | | | | | | | | |
| Test for overall effect: Z = 4.97 (P < 0.00001) | | | | | | | | |
| **59.1.2 < 60 ml/min/1.73 m2** | | | | | | | | |
| Bakris 2020 | 0.66 | 0.48 | 2711 | 0.95 | 0.69 | 2705 | 19.6% | -0.29 [-0.32, -0.26] |
| Tanaka 2025 | 0.5 | 0.45 | 49 | 0.73 | 0.65 | 48 | 7.9% | -0.23 [-0.45, -0.01] |
| **Subtotal (95% CI)** | | | **2760** | | | **2753** | **27.5%** | **-0.29 [-0.32, -0.26]** |
| Heterogeneity: Tau² = 0.00; Chi² = 0.27, df = 1 (P = 0.60); I² = 0% | | | | | | | | |
| Test for overall effect: Z = 18.06 (P < 0.00001) | | | | | | | | |
| **Total (95% CI)** | | | **7807** | | | **6705** | **100.0%** | **-0.36 [-0.44, -0.28]** |
| Heterogeneity: Tau² = 0.01; Chi² = 51.65, df = 7 (P < 0.00001); I² = 86% | | | | | | | | |
| Test for overall effect: Z = 8.86 (P < 0.00001) | | | | | | | | |
| Test for subgroup differences: Chi² = 1.68, df = 1 (P = 0.19), I² = 40.6% | | | | | | | | |

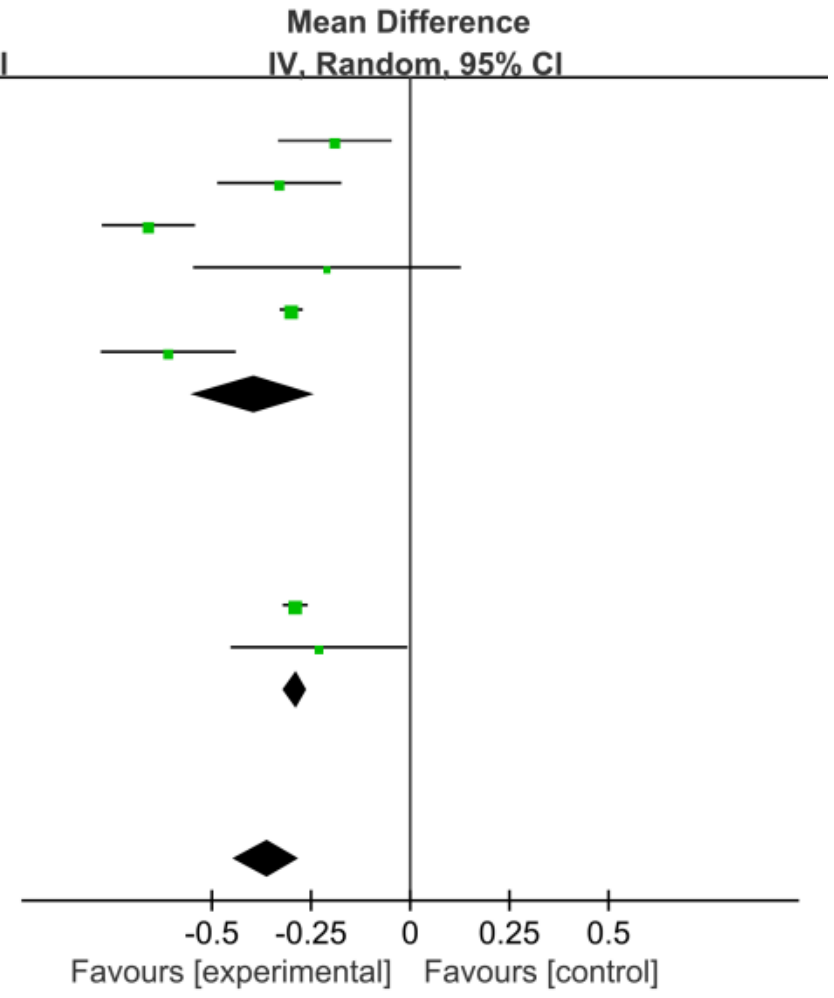


**Figure S19.** Subgroup analysis of the log-transformed LS mean ratio for UACR between the nsMRAs and control groups, stratified by baseline eGFR levels of participants.

| Study or Subgroup | Experimental Mean | Experimental SD | Experimental Total | Control Mean | Control SD | Control Total | Weight | Mean Difference IV, Random, 95% CI |
|---|---|---|---|---|---|---|---|---|
| **56.1.1 Finerenone** | | | | | | | | |
| Bakris 2015 | 0.75 | 0.55 | 718 | 0.94 | 0.68 | 94 | 12.3% | -0.19 [-0.33, -0.05] |
| Bakris 2020 | 0.66 | 0.48 | 2711 | 0.95 | 0.69 | 2705 | 19.6% | -0.29 [-0.32, -0.26] |
| Katayama 2017 | 0.85 | 0.46 | 83 | 1.06 | 0.57 | 12 | 4.4% | -0.21 [-0.55, 0.13] |
| Pitt 2021 | 0.62 | 0.45 | 3521 | 0.92 | 0.75 | 3476 | 19.7% | -0.30 [-0.33, -0.27] |
| Tanaka 2025 | 0.5 | 0.45 | 49 | 0.73 | 0.65 | 48 | 7.9% | -0.23 [-0.45, -0.01] |
| **Subtotal (95% CI)** | | | **7082** | | | **6335** | **63.9%** | **-0.29 [-0.31, -0.27]** |
| Heterogeneity: Tau² = 0.00; Chi² = 2.78, df = 4 (P = 0.60); I² = 0% | | | | | | | | |
| Test for overall effect: Z = 27.24 (P < 0.00001) | | | | | | | | |
| **56.1.2 Other nsMRAs agent** | | | | | | | | |
| Ito 2019 | 0.57 | 0.41 | 285 | 0.9 | 0.65 | 73 | 11.4% | -0.33 [-0.49, -0.17] |
| Ito 2020 | 0.42 | 0.3 | 222 | 1.08 | 0.85 | 227 | 14.1% | -0.66 [-0.78, -0.54] |
| Wada 2021 | 0.53 | 0.34 | 218 | 1.14 | 0.7 | 70 | 10.6% | -0.61 [-0.78, -0.44] |
| **Subtotal (95% CI)** | | | **725** | | | **370** | **36.1%** | **-0.54 [-0.74, -0.33]** |
| Heterogeneity: Tau² = 0.03; Chi² = 11.41, df = 2 (P = 0.003); I² = 82% | | | | | | | | |
| Test for overall effect: Z = 5.19 (P < 0.00001) | | | | | | | | |
| **Total (95% CI)** | | | **7807** | | | **6705** | **100.0%** | **-0.36 [-0.44, -0.28]** |
| Heterogeneity: Tau² = 0.01; Chi² = 51.65, df = 7 (P < 0.00001); I² = 86% | | | | | | | | |
| Test for overall effect: Z = 8.86 (P < 0.00001) | | | | | | | | |
| Test for subgroup differences: Chi² = 5.51, df = 1 (P = 0.02), I² = 81.9% | | | | | | | | |

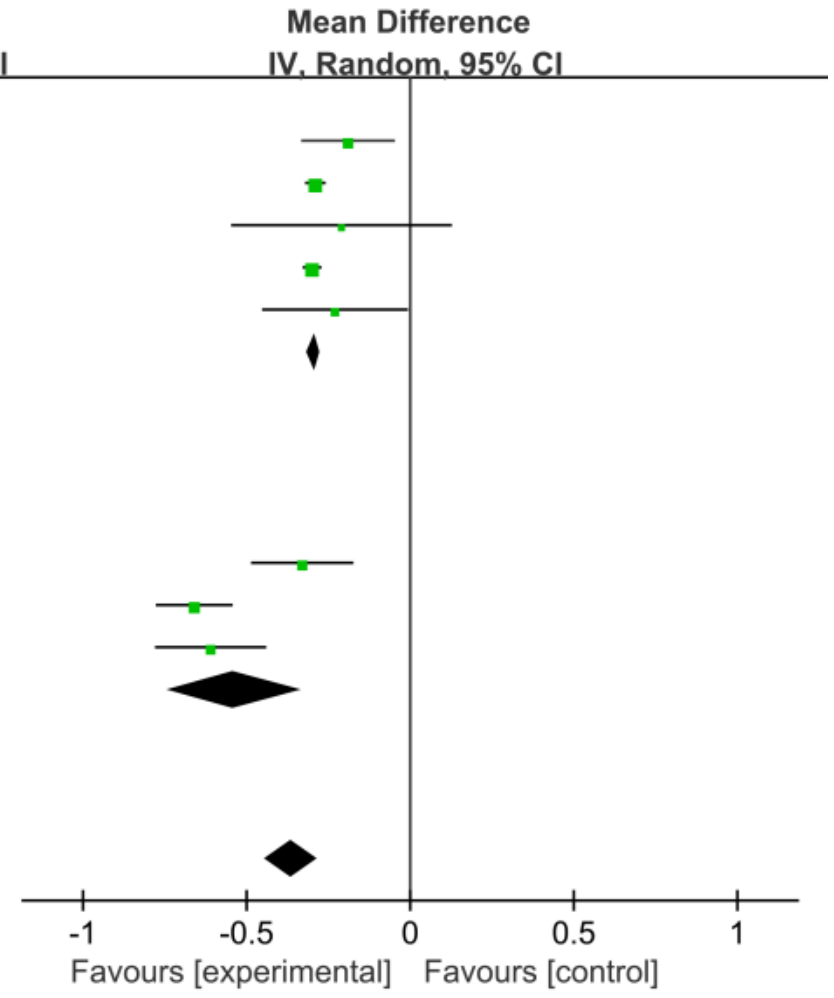


**Figure S20.** Subgroup analysis of the log-transformed LS mean ratio for UACR between the nsMRAs and control groups, stratified by drug type.

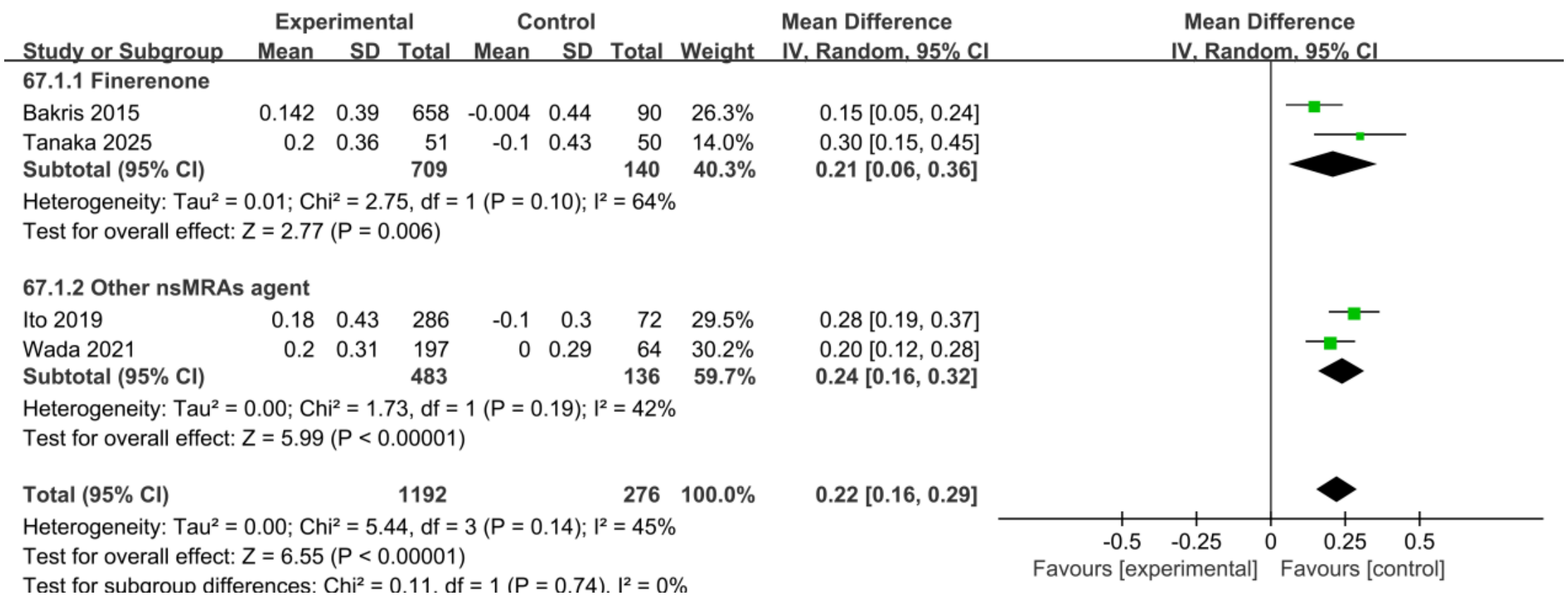


**Figure S21.** Subgroup analysis of change in serum potassium between the nsMRAs and control groups, stratified by drug type.

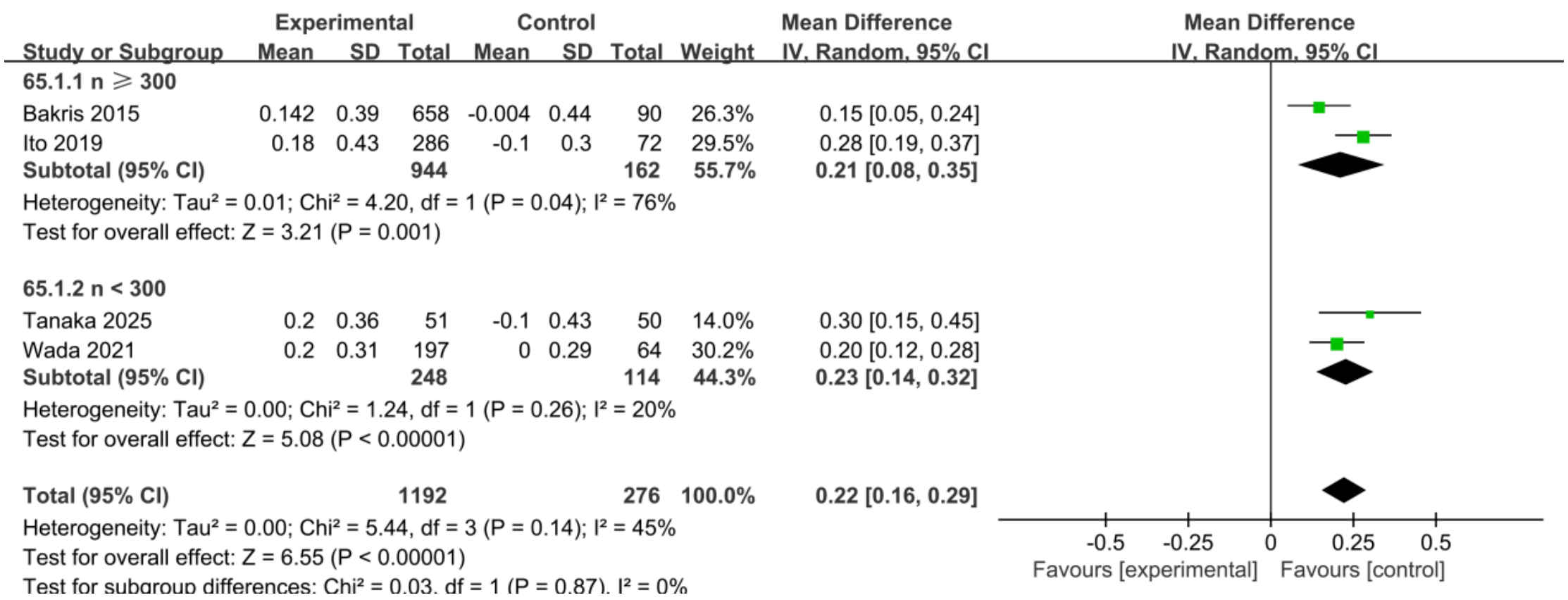


**Figure S22.** Subgroup analysis of change in serum potassium between the nsMRAs and control groups, stratified by sample size.

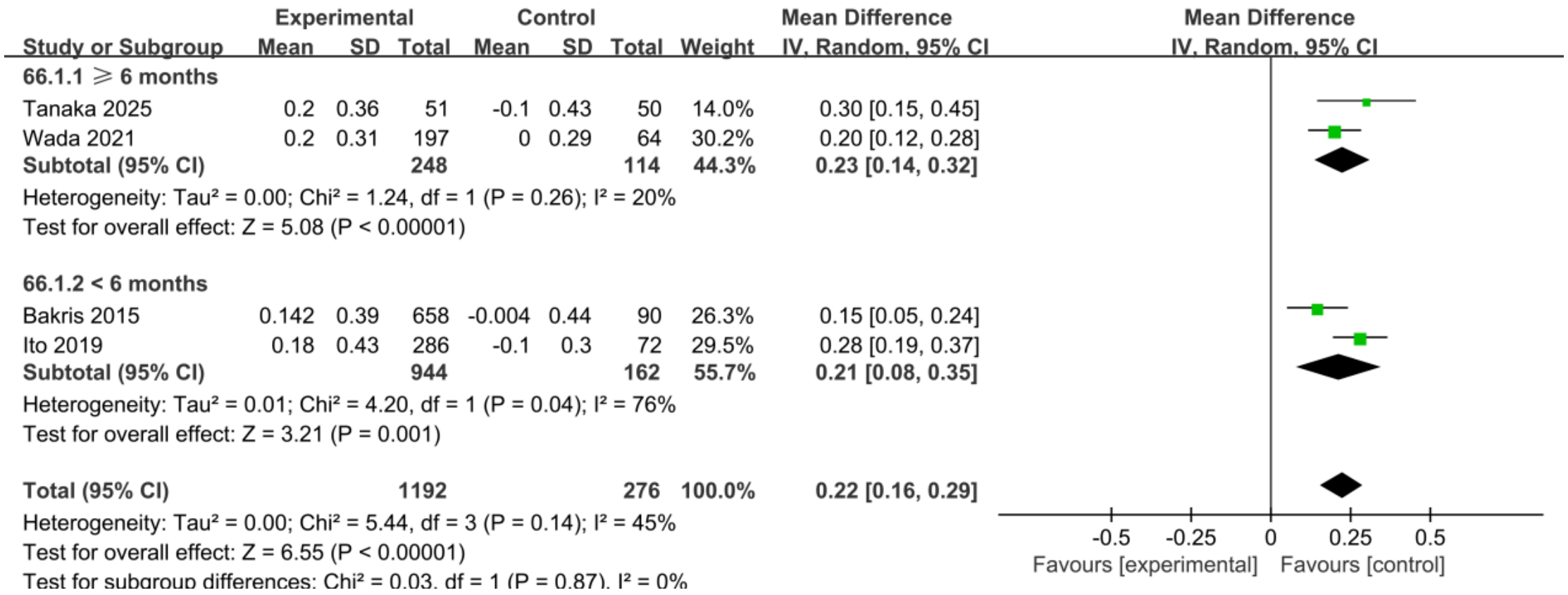


**Figure S23.** Subgroup analysis of change in serum potassium between the nsMRAs and control groups, stratified by study duration.

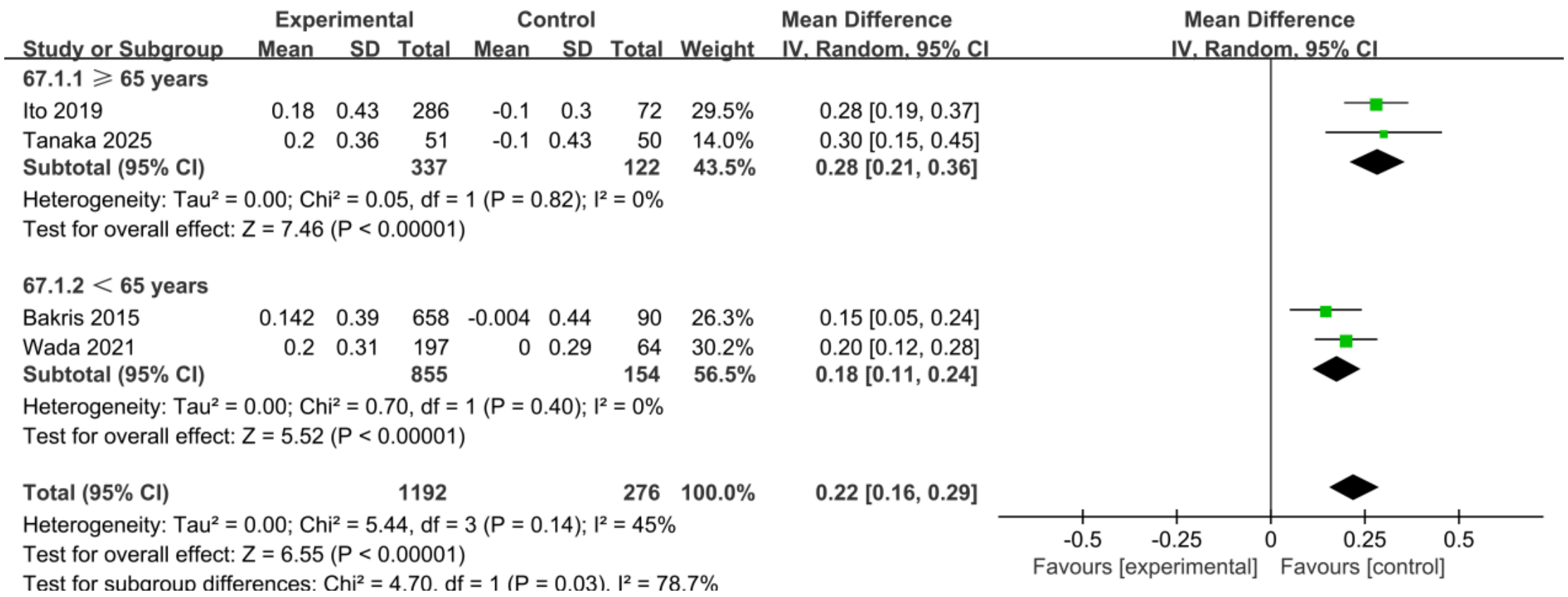


**Figure S24.** Subgroup analysis of change in serum potassium between the nsMRAs and control groups, stratified by age.
